# Redefining LiF as a Nanostructured Building Block for Interphase Engineering in Lithium Metal Batteries

**DOI:** 10.1002/advs.76108

**Published:** 2026-06-23

**Authors:** Gwangsik Kim, JinHyeok Cha

**Affiliations:** ^1^ School of Mechanical Engineering Chonnam National University Gwangju Republic of Korea

**Keywords:** dendrites, Ion transport, LiF‐rich SEI, lithium metal battery, molecular simulation, solid electrolyte interphase

## Abstract

Lithium metal batteries (LMBs) are regarded as promising next‐generation energy storage systems due to their high theoretical capacity and low reduction potential. However, their practical application is fundamentally limited by the instability of the solid electrolyte interphase (SEI) formed at the lithium metal–electrolyte interface. LiF‐rich SEIs have been widely proposed as an effective solution, as LiF provides strong electronic insulation that suppresses continuous electrolyte decomposition. Importantly, the performance of LiF‐rich SEIs is governed not simply by LiF content, but by the nanoscale structure, spatial distribution of LiF, and its heterogeneous interfaces with other SEI components. This review summarizes LiF‐rich SEI formation strategies based on electrolyte‐driven in situ processes and ex situ artificial coatings, and compares the structural and dynamic characteristics of the resulting interphases. Emphasis is placed on fluorinated electrolyte design using salts, additives, and solvents, which enables simultaneous electronic insulation and lithium‐ion transport through LiF nano structuring and interface engineering. Furthermore, LiF‐rich SEIs are redefined as assemblies of controllable nanoscale building blocks, highlighting the role of grain boundaries and heterogeneous interfaces. Computational approaches, including density functional theory, molecular dynamics, and machine‐learning‐based methods, are discussed as key tools for SEI engineering and predictive design.

## Graphical abstract

LiF‐rich solid electrolyte interphases in lithium metal batteries are redefined as controllable nanostructured building blocks rather than homogeneous protective films. This review shows how LiF distribution, grain boundaries, and heterogeneous interfaces govern electron blocking and Li ion transport, and compares fluorinated electrolytes, artificial coatings, and computational design principles.

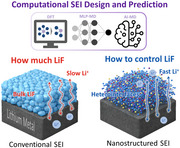

## 1 Introduction: LiF‐Rich SEI as a Central Strategy for Lithium Metal Anodes

The growing global demand for clean energy is accelerating electrification in the transportation sector and driving the development of robust grid‐scale energy storage systems, with lithium‐ion batteries (LIBs) at the core of this transformation [1, 2, 3]. LIBs have played a pivotal role in the portable electronics and electric vehicle (EV) markets, exhibiting rapid growth, with the global market size projected to exceed $69 billion by 2022 [4].

However, current LIBs technology has reached fundamental limitations in energy density [5], which constrains its ability to meet the long‐range driving and high‐capacity demands of the EV market that has expanded rapidly since the 2010s [4]. To overcome these limitations and achieve energy densities exceeding 400 Wh kg^−^^1^, lithium metal batteries (LMBs) employing lithium metal electrodes—which offer an exceptionally high theoretical capacity of 3860 mAh g^−^^1^—have attracted significant attention as promising next‐generation energy storage systems [2, 5, 6, 7]. In contrast to conventional LIBs that utilize graphite anodes, LMBs employ lithium metal as the anode active material [8]. Lithium metal possesses both an ultrahigh theoretical capacity of 3860 mAh g^−^^1^ and the lowest electrochemical potential of −3.04 V, enabling approximately ten times higher capacity compared to conventional LIB anodes [2, 9, 10, 11, 12, 13].

**FIGURE 1:**
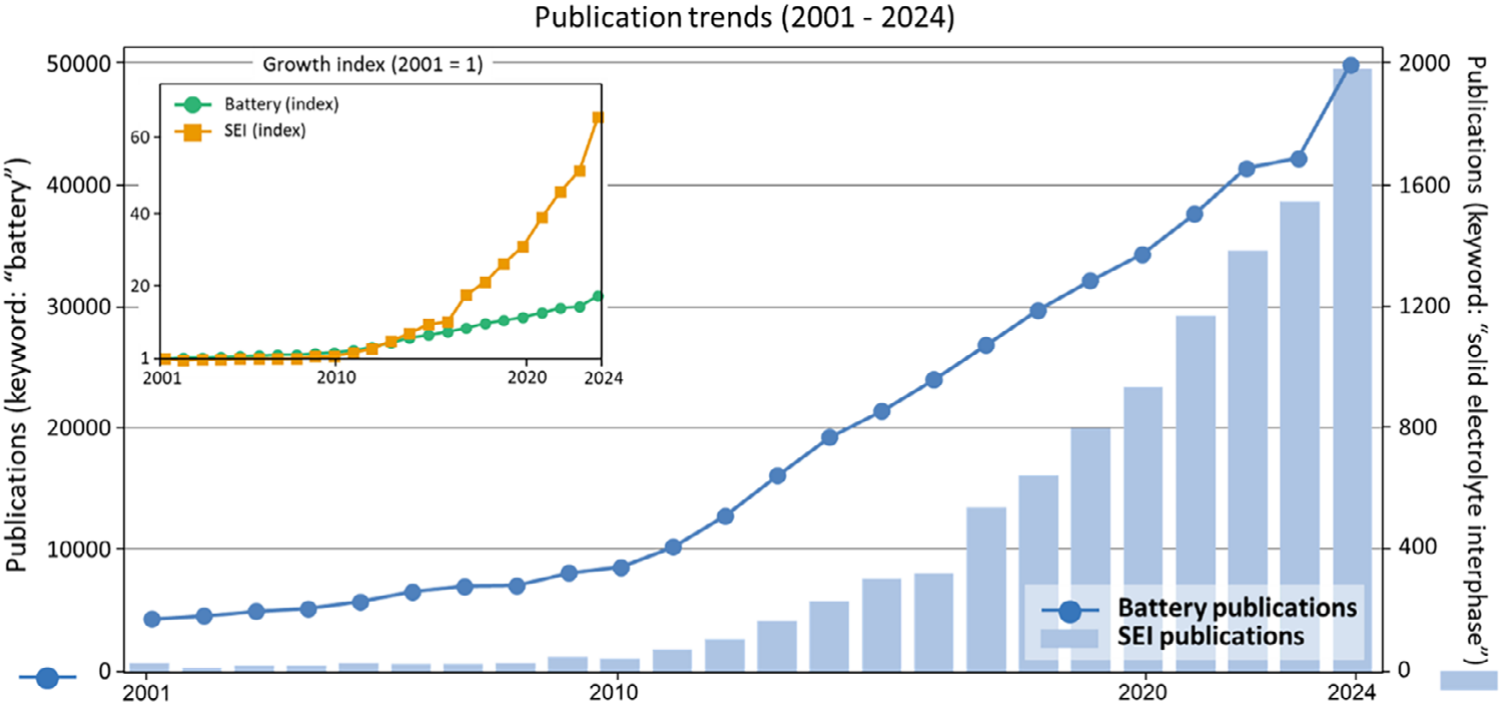
Annual number of publications related to batteries and solid electrolyte interphases (SEI). Publication statistics were obtained from the Web of Science Core Collection.

The most critical issue hindering the commercialization of lithium metal anodes is the formation of lithium dendrites during repeated plating/stripping cycles, accompanied by the formation of an unstable solid electrolyte interphase (SEI) [14, 15]. Owing to its high chemical reactivity and low electrochemical potential (−3.04 V), lithium metal spontaneously reacts with nearly all organic electrolytes upon contact, leading to SEI formation [16, 17, 18, 19, 20]. Although the SEI acts as a passivation layer that protects the lithium metal surface from further parasitic reactions, its intrinsic heterogeneity and mechanical fragility are primary factors responsible for dendrite formation [2, 21, 22]. The SEI generally exhibits a complex and heterogeneous mosaic structure composed of inorganic species (LiF, Li_2_O, Li_2_CO_3_) and organic components (ROCO_2_Li, ROLi) [23, 24, 25, 26]. This heterogeneity strongly influences the lithium‐ion deposition behavior [2]. Within the SEI, regions characterized by faster lithium‐ion transport or lower interfacial resistance act as electrochemical hot spots, where lithium‐ion flux becomes locally concentrated. At these hot spots, lithium preferentially nucleates and grows, resulting in dendritic structures [11, 12, 27, 28]. When dendrites propagate and bridge the gap between the anode and cathode, internal short circuits can occur, potentially triggering thermal runaway [12, 22, 29, 30, 31]. Consequently, stabilizing the lithium metal–electrolyte interface is widely recognized as the most critical technical challenge for the commercialization of LMBs [9, 18]. This trend underscores the growing recognition of interphase engineering as a central design principle for next‐generation lithium metal batteries (see Figure 1).

The SEI formed at the interface between lithium metal and the electrolyte is a key determinant of lithium metal stability [14, 32, 33]. An ideal SEI must completely block electron transport while simultaneously allowing smooth and efficient migration of lithium ions (Li^+^) [18, 34, 35, 36]. However, naturally formed SEIs are intrinsically heterogeneous and mechanically brittle, undergoing repeated fracture and reformation during charge–discharge cycling [20, 23, 35]. This continuous breakdown and regeneration process leads to persistent consumption of active lithium and electrolyte, resulting in severe degradation of battery lifespan and safety, as well as a reduction in Coulombic efficiency (CE) [18, 30, 36, 37, 38]

Inorganic components such as Li_2_O and Li_3_N have also been widely identified in the SEI [39, 40]. Owing to its relatively low Li^+^ diffusion barrier, Li_2_O can facilitate Li^+^ transport and improve the initial Li nucleation behavior [41]. Likewise, Li_3_N can promote uniform Li electrodeposition because of its high Li^+^ conductivity and lithiophilic nature.

However, although these components are beneficial for specific functions, they have intrinsic limitations in simultaneously meeting the stringent requirements of chemical stability, mechanical robustness, low solubility, and long‐term interfacial stability under practical operating conditions [42, 43]. While Li_2_O enables fast Li^+^ migration, it is less effective in providing sufficient chemical stability and mechanical protection to suppress continuous parasitic reactions with the electrolyte [6]. Li_3_N exhibits high ionic conductivity and lithiophilicity in the bulk state, but its lower ionic conductivity at the SEI/Li interface and insufficient electron‐blocking capability significantly limit its ability to prevent continuous electrolyte decomposition [44, 45]. As a result, maintaining Li_3_N as a stable interphase component over extended cycling in practical electrolyte environments remains challenging [46]. Therefore, although these inorganic species can complement specific SEI functionalities, they are insufficient as a comprehensive interfacial stabilization strategy that must simultaneously satisfy the requirements of electron‐blocking capability, facile Li^+^ transport, chemical stability, and mechanical durability at the Li metal interface.

A key strategy to mitigate these challenges involves the formation and regulation of a LiF‐rich SEI, namely a lithium fluoride–rich solid electrolyte interphase [28, 47, 48, 49]. LiF exhibits a wide bandgap of approximately 13.6 eV, enabling it to function as an effective electronic insulator that suppresses electron tunneling [11, 28]. Owing to its small ionic constituents, LiF possesses the shortest interionic spacing among SEI components, leading to an exceptionally high lattice energy of 1036 kJ mol^−^^1^, which fundamentally underpins its high mechanical strength [20, 50]. In addition, the high surface energy of LiF favors uniform and dendrite‐free lithium deposition by lowering the surface diffusion barrier for lithium ions [24, 28].

**FIGURE 2:**
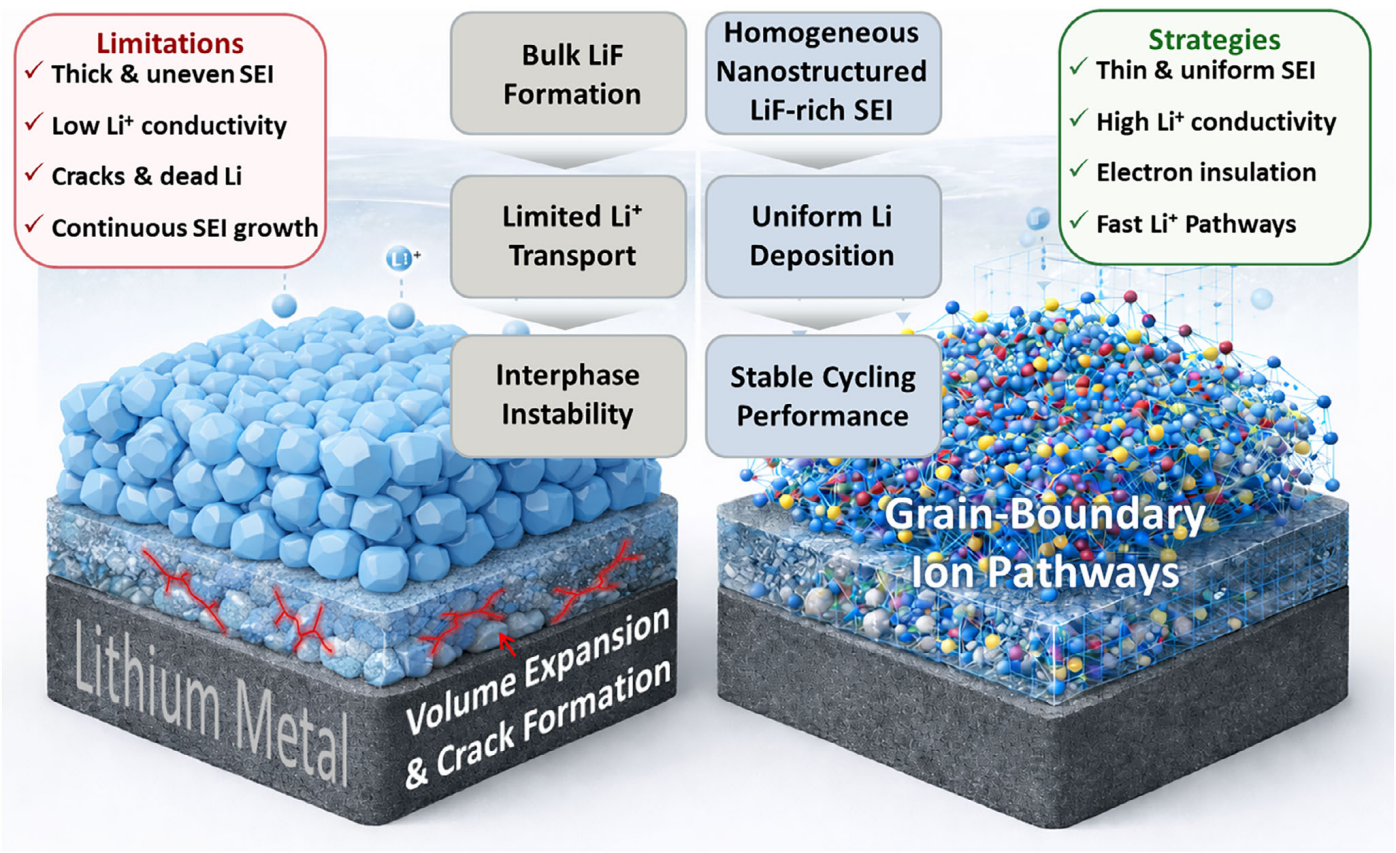
Schematic comparison of LiF‐rich SEIs governed by bulk properties versus nano structural and interfacial control.

Although increasing LiF content is widely acknowledged as essential for interfacial stabilization in LMBs, simply increasing the amount of LiF is insufficient to ensure optimal performance. Instead, the physical properties and spatial distribution of LiF within the SEI play decisive roles. If LiF precipitates outside the compact SEI region that constitutes the core protective layer, it cannot effectively regulate lithium electrode dissolution [51]. Moreover, owing to its high ionic migration barrier, excessively concentrated or non‐uniformly distributed LiF can impede lithium‐ion transport and induce large overpotentials [36, 52, 53]. When LiF is finely dispersed and intimately mixed with other SEI components, lithium‐ion transport can be enhanced through heterointerfaces formed between LiF and adjacent phases [53]. Therefore, the distribution, thickness, grain size, and nanostructure of LiF are key parameters governing the balance between electronic insulation and ionic conductivity within the SEI [6, 24, 53]. An overly thick LiF layer increases interfacial resistance by hindering ion transport, whereas a thin or structurally non‐uniform LiF layer allows electron leakage, thereby compromising SEI stability [16, 28, 36, 45, 54, 55, 56]. Accordingly, the essence of SEI design lies not in “how much LiF is formed,” but in “how LiF is controlled into the desired structure” (see Figure 2) [36, 37, 57].

Recent studies increasingly emphasize strategies focused on controlling the nanoscale structure and heterogeneous interfaces of LiF, rather than relying solely on its bulk properties [36, 52, 53, 56]. Uniformly dispersed nanocrystalline LiF can preserve electronic insulation and mechanical robustness while simultaneously providing Li^+^ diffusion pathways through interfaces with other inorganic components, such as Li_2_O and Li_3_N [30, 45, 54, 58]. Such composite architectures have been proposed as a fundamental solution for simultaneously maximizing electronic insulation and ionic conductivity in the SEI [20, 23, 53].

Consequently, research on interfacial stabilization in LMBs has advanced beyond simple discussions regarding the presence or absence of LiF. Instead, it is now redefining the trade‐off between electronic insulation and ionic conductivity through deliberate nano structural control of LiF‐rich SEIs [36, 57, 58]. Establishing such control strategies is essential for addressing fundamental challenges in LMBs, including lithium dendrite growth, low Coulombic efficiency, and electrolyte depletion, and is expected to represent a decisive step toward the commercialization of high‐energy, high‐safety lithium metal batteries [9, 49, 56].

## 2 Formation of LiF‐Rich SEI

SEI formation is not a static process in which a uniform protective film simply covers the electrode surface; rather, it is a highly rapid and complex out‐of‐equilibrium interfacial reaction initiated immediately upon contact between lithium metal and the electrolyte [59, 60]. When the Fermi level of lithium metal lies above the lowest unoccupied molecular orbital (LUMO) of the electrolyte components, electrons are transferred from the electrode to the electrolyte, thereby triggering the reductive decomposition of solvents and lithium salts [61]. Because electrolyte components differ in their reduction potentials, bond dissociation energies, surface adsorption characteristics, and Li^+^ coordination environments (solvation structures), they decompose through distinct pathways and with different reaction kinetics [62]. As a result, the initial SEI does not form as a uniform continuous film, but instead begins through island‐like nucleation, in which decomposition products are preferentially deposited at surface defects, high‐curvature regions, or sites with strong local electric fields and pronounced electron tunneling [59].

As these initial nuclei subsequently undergo growth, agglomeration, and coalescence, the SEI evolves into a thin interfacial layer composed of mixed organic and inorganic decomposition products. Throughout this process, the composition and nanostructure of the SEI dynamically evolve in response to local potential, electrolyte composition, Li^+^ flux, and mechanical stress [60, 63]. Once the SEI reaches a thickness of a few nanometers, electron transfer is progressively blocked, thereby limiting further electrolyte reduction [64, 65]. However, during prolonged charge/discharge cycling, the large volume changes associated with lithium plating and stripping induce repeated cracking, dissolution, densification, and reformation of the SEI [66].

The commercialization of lithium metal batteries critically depends on the stability of the SEI formed on the lithium metal surface [38]. An uneven and unstable SEI induces lithium dendrite growth, continuous electrolyte depletion, and low Coulombic efficiency (CE), thereby severely compromising battery lifespan and safety. A fluorination strategy—employing fluorine (F)‐containing compounds in the electrolyte to generate a LiF‐rich inorganic SEI on the lithium metal surface—has therefore emerged as a key approach [16, 20].

The fundamental principle underlying the fluorination strategy is thermodynamic selectivity [16, 67]. Fluorine‐containing species, including lithium salts, additives, and solvents, generally exhibit low LUMO energy levels [27, 45, 68, 69]. As a result, these species preferentially undergo reductive decomposition at the extremely low reduction potential of lithium metal (−3.04 V) relative to other electrolyte components [23, 69]. This selective reduction process leads to the formation of LiF, a thermodynamically stable inorganic compound, on the lithium metal surface [25, 70, 71].

**FIGURE 3:**
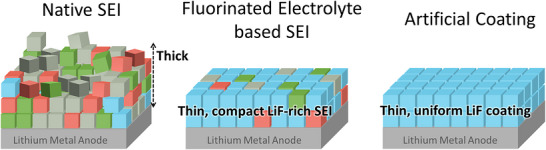
Schematic illustration of different formation pathways of LiF‐containing SEI on lithium metal anodes. The left panel shows an SEI formed through conventional electrolyte decomposition during cycling. The middle panel depicts an SEI formed in situ using fluorinated electrolytes, where fluorine‐containing species participate in interfacial reactions during SEI formation. The right panel represents an ex situ LiF layer formed via artificial coating strategies prior to electrochemical cycling.

Fluorination strategies can be broadly classified into two main approaches: in situ formation of a LiF‐rich SEI through the use of fluorinated salts, additives, or solvents in the electrolyte, and ex situ construction of a LiF‐rich protective layer via direct coating of the lithium metal surface (see Figure 3) [11, 20, 39, 72]. Accordingly, this section analyzes these two implementation strategies, with particular emphasis on the roles and mechanisms of various fluorinated electrolyte components, as well as the strategies and advantages associated with artificial LiF layer construction.

### 2.1 Formation of LiF‐Rich SEI via Fluorinated Electrolytes

#### 2.1.1 Properties and Limitations of Conventional Fluorinated Salt: LiPF_6_

**FIGURE 4:**
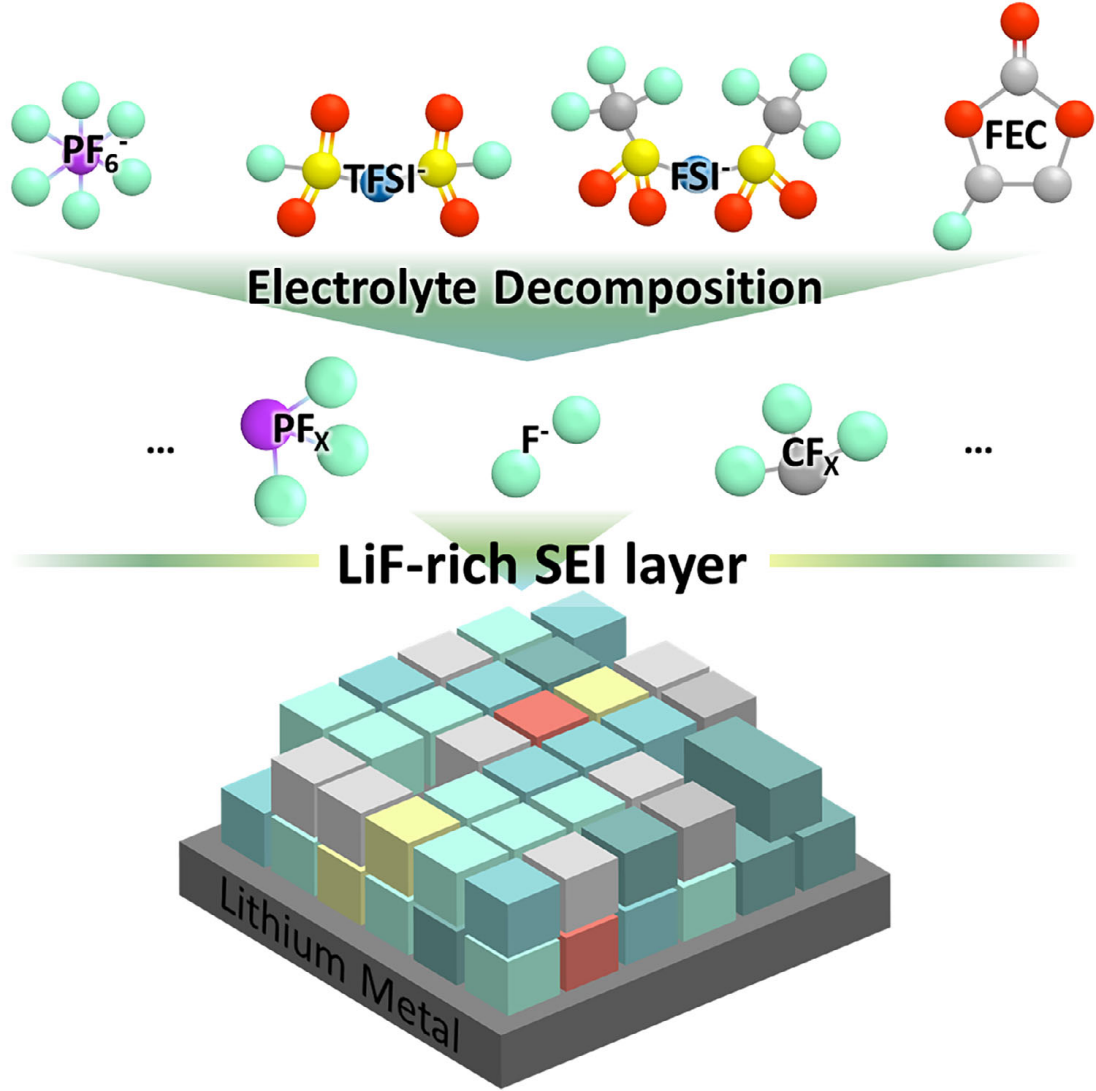
Schematic illustration of LiF‐rich solid electrolyte interphase (SEI) formation via fluorinated electrolytes. Representative fluorinated electrolyte components—including PF_6_^−^, TFSI^−^, FSI^−^ anions and the fluorinated solvent/additive fluoroethylene carbonate (FEC)—are shown above the electrolyte phase. During electrochemical operation, these fluorine‐containing species participate in interfacial reactions at the lithium metal surface, contributing to the formation of a fluorine‐containing SEI layer on the lithium metal anode.

The anion of the lithium salt used in the electrolyte plays a critical role in determining the chemical composition and physical properties of the SEI (see Figure 4) [1, 34, 36]. In particular, fluorine‐containing anions provide the most direct route for SEI control, as they can be directly reduced at the lithium metal surface to form LiF [36, 56, 73, 75]. LiPF_6_ is currently widely used lithium salt in commercial lithium‐ion batteries. This widespread adoption does not stem from superiority in any single property, but rather from its ability to provide an optimal balance among multiple essential characteristics [22, 76, 77].

LiPF_6_ exhibits one of the highest ionic conductivities in nonaqueous electrolytes based on mixed alkyl carbonate solvents [22]. Although its ionic conductivity in ethylene carbonate(EC)/dimethyl carbonate(DMC) is lower than that of LiAsF_6_, and it possesses a lower dissociation constant than imide‐based salts and lower ionic conductivity than LiBF_4_, LiPF_6_ effectively balances these competing requirements. LiPF_6_ contributes to the formation of two critical protective features at the electrode–electrolyte interface. First, it provides high oxidative stability, resisting oxidation up to 5.1 V in mixed carbonate electrolytes [78]. Consequently, it is among the few lithium salts capable of supporting high‐voltage cathode operation (∼4.0 V) [76]. Second, LiPF_6_ effectively passivates the aluminum current collector at elevated potentials. Whereas salts such as LiAsF_6_, LiSbF_6_, and LiTf induce severe aluminum corrosion, LiPF_6_ maintains aluminum stability even at potentials exceeding 4.2 V [22, 79, 80].

**FIGURE 5:**
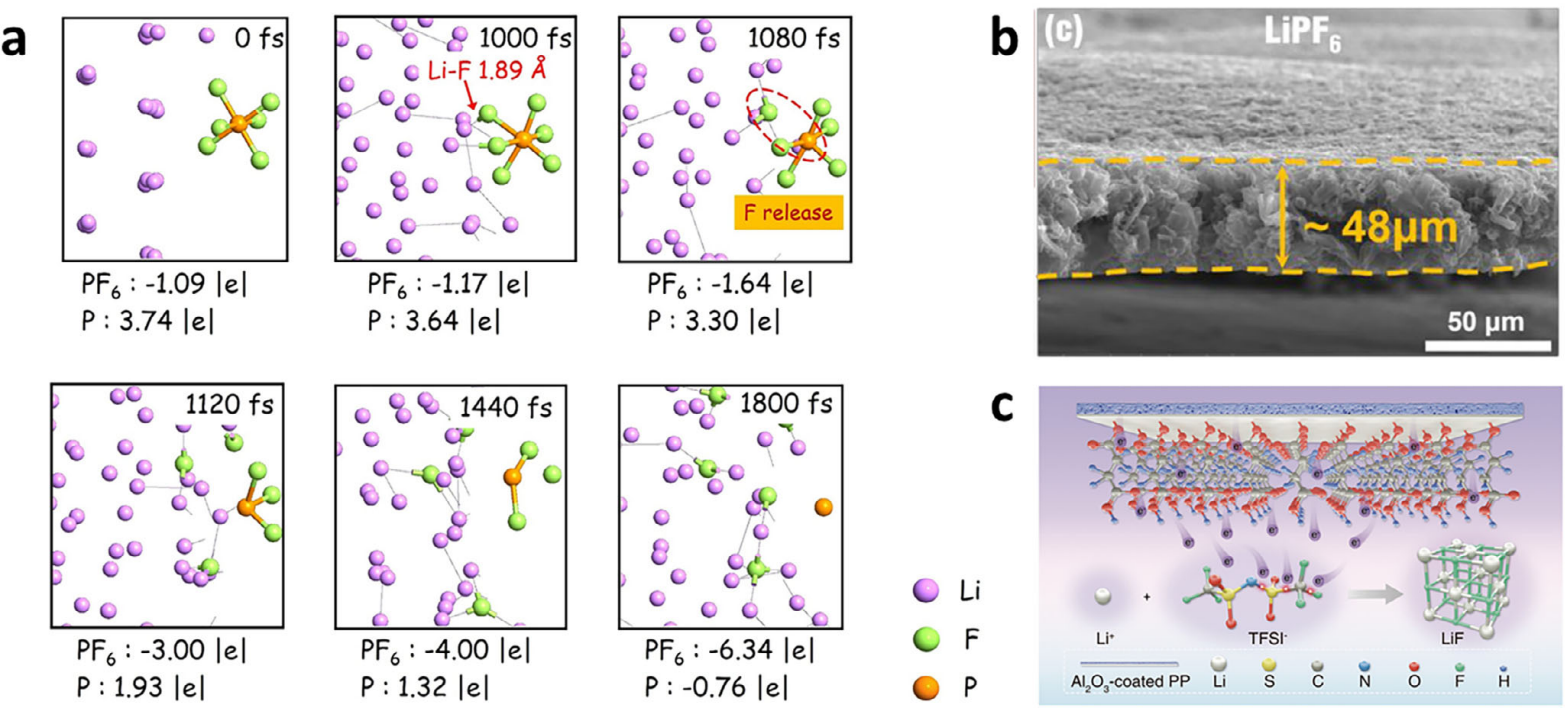
(a) Ab initio molecular dynamics simulation snapshots illustrating the reductive decomposition of LiPF_6_ at a Li(110) surface. The PF_6_^−^ anion undergoes rapid P─F bond cleavage within the picosecond timescale, accompanied by electron transfer from the lithium metal and subsequent formation of LiF and PF_5_ at the interface. Reproduced with permission [73]. Copyright 2023, Elsevier. (b) Cross‐sectional scanning electron microscopy (SEM) image of the surface layer formed in a LiPF_6_‐based carbonate electrolyte, showing the thick interfacial deposit generated on the lithium metal electrode during electrochemical cycling. Reproduced with permission [74]. Copyright 2022, American Chemical Society. (c) Schematic representation of interfacial chemistry induced by TFSI^−^‐based electrolytes, illustrating how interfacial dipole modulation promotes preferential bond cleavage within the TFSI^−^ anion and facilitates LiF nanocrystal formation at the lithium metal surface. Reproduced with permission [27]. Copyright 2022, American Association for the Advancement of Science.

Ab initio molecular dynamics (AIMD) simulations reveal that LiPF_6_ decomposes rapidly at the lithium metal surface. Upon contact with the Li(110) surface, the PF_6_^−^ anion undergoes P─F bond cleavage within 1080 fs, generating PF_5_ and LiF (Figure 5a). During this process, the PF_6_^−^ anion accepts a total of five electrons from the lithium metal within 2 ps, and the Bader charge of the central phosphorus atom decreases sharply from +3.74 to −0.76. These results quantitatively demonstrate that LiPF_6_ undergoes deep reductive decomposition at the lithium surface, leading to LiF formation [73].

Despite these advantages, LiPF_6_ exhibits critical limitations in lithium metal batteries and in high‐temperature or humid environments, primarily due to the intrinsic instability of the PF_6_^−^ anion. The most severe drawback of LiPF_6_ is its low thermal stability and high susceptibility to hydrolysis. Owing to the weak P─F bond, LiPF_6_ readily decomposes into LiF and the toxic gas PF_5_ [22]. Thermogravimetric analysis (TGA) indicates that LiPF_6_ loses approximately 50% of its mass at 200°C under dry conditions, while significant degradation occurs even at temperatures as low as 70°C in aqueous environments. In the presence of trace amounts of water (H_2_O), LiPF_6_ can trigger a chain hydrolysis reaction that generates lethal hydrogen fluoride (HF) [81, 82, 83]. In addition, LiPF_6_ dissociates or decomposes under thermal or external stimuli to form LiF and the strong Lewis acid PF_5_, with this reaction reaching a dynamic equilibrium [81, 84].

As the temperature increases, the decomposition of LiPF_6_ is accelerated, leading to enhanced formation of LiF and PF_5_. Interactions with electrolyte solvent molecules further shift this equilibrium toward PF_5_ generation [85]. The produced PF_5_ subsequently reacts with residual moisture in the electrolyte to generate additional HF [84]. The rate constant for the reaction between H_2_O and LiPF_6_ in 1M LiPF_6_ EC/ethyl methyl carbonate(EMC)) electrolyte increases with temperature, demonstrating that HF generation in LiPF_6_‐based electrolytes is strongly temperature dependent and becomes particularly severe under high‐temperature operating conditions [81]. The generated HF causes extensive corrosion and performance degradation throughout the electrolyte, cathode, and anode [76, 80, 86]. HF induces the formation of an unstable SEI on the lithium metal surface, resulting in highly non‐uniform lithium plating. For example, lithium deposition for 10 h at a current density of 0.3 mA cm^−^^2^ in a commercial LiPF_6_ electrolyte (EC:diethyl carbonate(DEC)) produces a lithium layer approximately 48 µm thick, which is more than three times thicker than the theoretical lithium thickness of 15 µm expected for the same capacity (Figure 5b) [74]. This excessive thickness reflects severe dead lithium accumulation and dendritic growth originating from unstable interfaces formed by decomposition products such as HF [9, 35, 87].

To enable the practical application of LiPF_6_‐based fluorinated electrolytes in high‐energy cells, additional electrolyte design is required to suppress HF generation and gas evolution while preserving the advantage of forming a LiF‐rich SEI [88].

One effective strategy is the introduction of multifunctional additives with HF‐scavenging capability [89]. Additives such as trimethoxy(3,3,3‐trifluoropropyl)silane (TTS) can chemically remove acidic byproducts by directly reacting with HF in the electrolyte to form Si─F bonds [45]. This reaction mitigates HF‐induced cathode surface corrosion and transition metal dissolution, while simultaneously alleviating the continuous degradation of the anode SEI. Moreover, because additives such as TTS can preferentially decompose at both cathode and anode interfaces to form stable interphases, they can serve not merely as HF scavengers but also as full‐cell‐level interphase‐stabilizing additives.

Another strategy is to design a hybrid electrolyte that localizes fluorination reactivity only at the interfacial regions where LiF formation is required, rather than excessively fluorinating the entire electrolyte [90]. Excessive use of fluorinated solvents can lead to several drawbacks, including high cost, increased viscosity, poor wettability, and severe gas evolution [91]. Accordingly, localized high‐concentration electrolytes (LHCEs) and hybrid electrolyte strategies have recently attracted considerable attention [41]. In these systems, the bulk electrolyte consists of non‐fluorinated or weakly fluorinated solvents with low viscosity and low gas evolution, whereas fluorinated components are strategically confined to the Li^+^ solvation sheath or interfacial reaction regions [90]. Such designs can preserve the ability to form a LiF‐rich SEI/cathode electrolyte interphase (CEI) while minimizing excessive electrolyte decomposition and gas evolution.

#### 2.1.2 LiTFSI‐Based Electrolytes: Stability and Limited LiF Formation

To overcome the limitations associated with LiPF_6_, imide‐based lithium salts such as lithium bis(trifluoromethanesulfonyl)imide(LiTFSI) and lithium bis(fluorosulfonyl)imide(LiFSI) have emerged as promising alternatives [76]. These salts exhibit high thermal stability and excellent ionic conductivity, rendering them promising candidates for next‐generation electrolyte systems [22, 45].

LiTFSI possesses a lower LUMO energy than LiFSI or 1,2‐dimethoxyethane(DME), making it more susceptible to reduction reactions at the lithium metal surface [16]. A key advantage of LiTFSI is its exceptional thermal stability, with decomposition occurring only at temperatures as high as 360°C [76]. This behavior contrasts sharply with that of LiPF_6_, which can undergo degradation even at temperatures around 70°C in solution [22]. Despite this advantage, LiTFSI generally forms an SEI with a relatively lower LiF content compared to LiFSI [54]. The TFSI^−^ anion predominantly decomposes via C─S bond cleavage, a pathway associated with a relatively high activation energy of 0.30 eV. As a result, LiTFSI undergoes less complete reduction, leading to the preferential formation of organofluorine species, such as –CF_3_ groups, on the SEI surface rather than LiF [36]. It has been shown that LiFSI primarily decomposes via S─F bond cleavage, while LiTFSI tends to initiate decomposition through C─S or S─N bond cleavage pathways [92].

LiTFSI also suffers from the drawback of corroding aluminum (Al) current collectors, and is therefore commonly used in combination with other salts, such as LiFSI or lithium nitrate (LiNO_3_), rather than as a single‐salt electrolyte [36]. Although LiTFSI alone may exhibit relatively low Coulombic efficiency due to its limited ability to generate LiF, previous studies have demonstrated synergistic effects when LiTFSI is combined with LiFSI [45]. In Li/Cu cell tests employing a mixed‐salt electrolyte consisting of LiTFSI and LiFSI (4.6 M LiFSI + 2.3 M LiTFSI in DME), a Coulombic efficiency of 97.9% was achieved at a current density of 0.5 mA cm^−^^2^ [45]. Moreover, the incorporation of LiFSI significantly reduced the overpotential at high current densities (10 mA cm^−^^2^). X‐ray photoelectron spectroscopy (XPS) analysis confirmed that the SEI formed in this dual‐salt system contained a high proportion of inorganic fluorine‐ and oxygen‐containing species.

A representative strategy for overcoming the limitations of single‐salt LiTFSI electrolytes is the design of dual‐salt electrolytes in combination with LiFSI [93]. In such dual‐salt systems, Li^+^ is not coordinated by a single type of anion; instead, both TFSI^−^ and FSI^−^ simultaneously participate in the primary solvation shell, forming a mixed coordination structure [94]. In other words, Li^+^ migrates through a solvation sheath co‐constructed by TFSI^−^ and FSI^−^, and this mixed coordination environment directly affects not only Li^+^ transport behavior, but also anion decomposition pathways and the resulting SEI composition [93]. In particular, FSI^−^ interacts strongly with Li^+^, thereby promoting anion‐derived decomposition and contributing to the formation of a dense, inorganic‐rich, and LiF‐rich SEI. In addition, LiFSI‐based electrolytes can form a ligand‐channel‐like structure that facilitates Li^+^ migration, thus simultaneously enhancing ion‐transport properties and interfacial reactivity [95].

By contrast, TFSI^−^, which has a relatively large molecular structure and contains a ‐CF_3_ group, exhibits solvation and decomposition behavior distinct from that of FSI^−^ [54, 94]. Although a LiTFSI‐rich composition can restrict solvent and ion mobility and increase viscosity, TFSI^−^ provides high thermal, chemical, and oxidative stability, thereby complementing the overall stability of the electrolyte [79, 96]. Therefore, within the dual‐salt system, FSI^−^ primarily promotes Li^+^ transport and LiF‐rich SEI formation, whereas TFSI^−^ plays a complementary role in maintaining the structural, high‐temperature, and high‐voltage stability of the electrolyte [93]. This combination is particularly meaningful because it mitigates the high viscosity and interfacial instability that may arise when LiFSI is used alone, while preserving the high ionic conductivity and LiF‐forming capability associated with LiFSI [80, 91]. Consequently, the LiTFSI/LiFSI dual‐salt electrolyte represents a more versatile electrolyte design strategy than single‐salt electrolytes, as it can simultaneously regulate the solvation structure, interphase composition, ion‐transport properties, and electrode stability.

For example, an electrolyte prepared by mixing 0.5 M LiTFSI and 0.5 M LiFSI in 1,3‐dioxolane(DOL)/DME formed an SEI containing both LiF and CF_3_‐derived components and achieved a Coulombic efficiency of 99% [45]. In addition, LiTFSI can be diluted using weakly coordinating fluorinated ethers, such as BTFE (bis(2,2,2‐trifluoroethyl) ether), enabling Coulombic efficiencies comparable to those of high‐concentration electrolytes (HCEs) while reducing the overall salt concentration [36].

An alternative strategy involves the design of novel lithium salts that retain the fundamental structural framework of the LiTFSI anion while improving chemical stability and lithium‐ion coordination characteristics. Selectively Fluorinated Aromatic Lithium Salts(SFALS) derivatives derived from LiTFSI have been synthesized by substituting the –CF_3_ group with fluorinated aromatic moieties. Among these, (3,5‐difluorobenzenesulfonyl)(trifluoromethanesulfonyl)imide(LiDF) exhibits a significantly lower LUMO energy (1.59 eV) than TFSI^−^ (4.38 eV), rendering it more readily reducible. The use of LiDF enables a high critical current density of 1 mA cm^−^^2^ and supports stable cycling for over 700 h in a Li/Li symmetric cell, in stark contrast to LiTFSI‐based systems, which short‐circuited after only 94 h [97].

Recent studies have explored innovative interfacial engineering approaches to overcome the limitations of LiTFSI. Liu, Yujing, et al. introduced self‐assembled monolayers (SAMs) at the electrode interface to modulate the surface dipole moment, thereby promoting C─F bond cleavage in LiTFSI and inducing the formation of LiF nanocrystals (Figure 5c) [27]. Symmetric cells incorporating SAM‐modified interfaces exhibited stable cycling for more than 1000 cycles (exceeding 2500 h) while maintaining a low overpotential of approximately 40 mV, whereas cells employing pristine Al_2_O_3_ interfaces displayed a higher overpotential of 75 mV after only 700 h.

#### 2.1.3 LiFSI‐Based Electrolytes: Efficient LiF Formation and SEI Reinforcement

LiFSI exhibits superior chemical stability and thermal robustness compared to LiPF_6_ [45]. In addition, LiFSI shows significantly higher solubility in carbonate‐based solvents than LiPF_6_. While the saturation solubility of LiPF_6_ in EC/DMC (1:1) mixtures is below 5 M, LiFSI demonstrates solubility exceeding 10 M [99]. This high solubility enables the design of high‐concentration electrolytes, which are critical for enhancing LMB performance.

**FIGURE 6:**
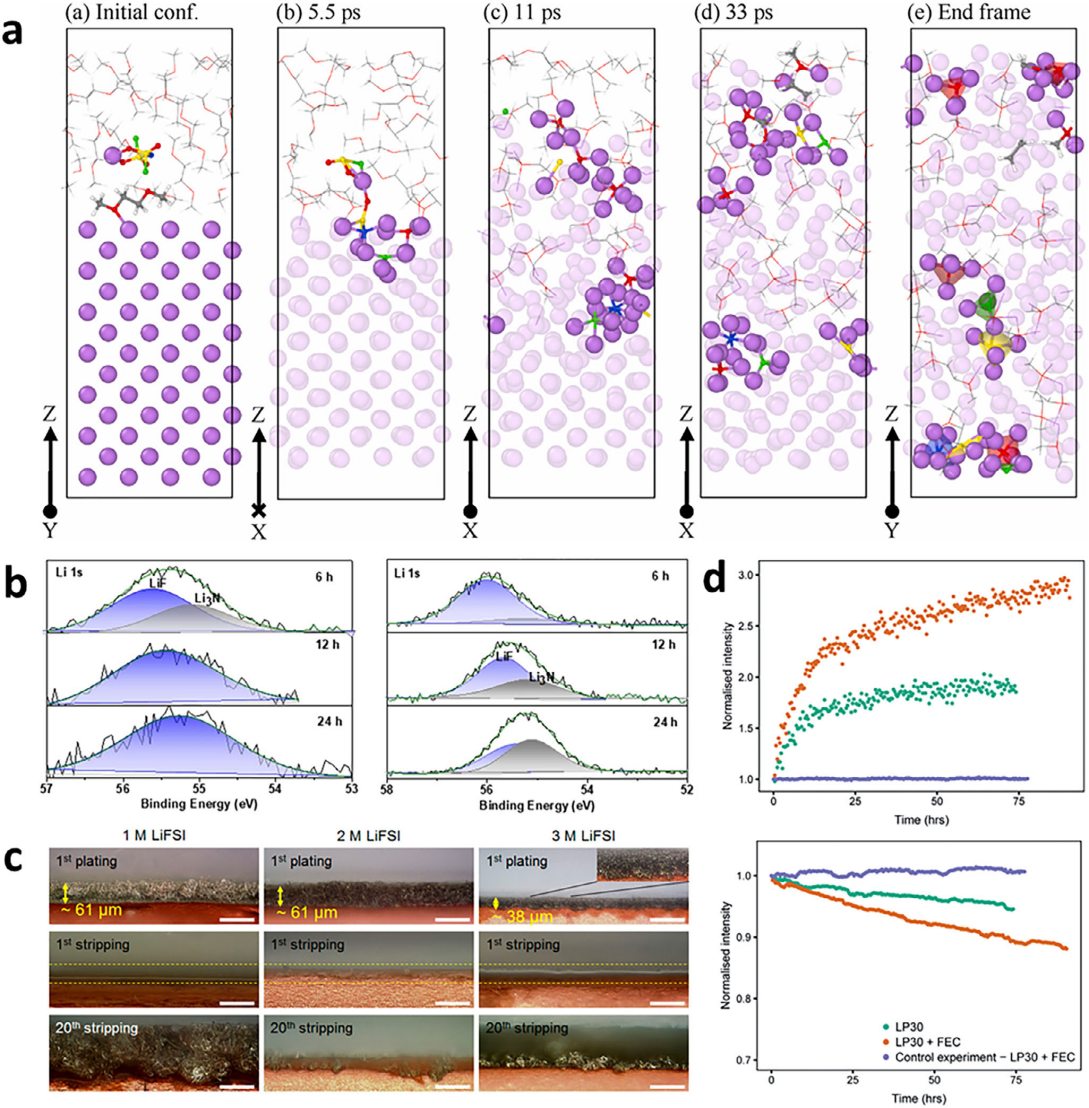
(a) Reactive molecular dynamics simulation snapshots illustrating the early‐stage decomposition of the LiFSI anion at the lithium metal interface and the subsequent formation of inorganic SEI species during the first tens of picoseconds. Reproduced with permission [1]. Copyright 2022, Elsevier. (b) X‐ray photoelectron spectroscopy (XPS) analysis comparing the chemical composition of SEI layers formed in 1 M LiFSI/DME and 1 M LiTFSI/DME electrolytes after immersion at the lithium metal surface. Both SEIs consist of inorganic species (e.g., LiF and Li_3_N) and organic components (e.g., ROCO_2_Li and ROLi); however, their relative compositions differ markedly. In LiTFSI/DME, Li_3_N dominates the inorganic fraction after 24 h of immersion, whereas Li_3_N appears only after 6 h in LiFSI/DME. Reproduced with permission [16]. Copyright 2020, Elsevier. (c) Morphological and compositional evolution of the SEI formed in a DME‐based LiFSI electrolyte as a function of salt concentration (1–3 m). SEM and XPS analyses reveal that increasing the LiFSI concentration leads to the formation of a thicker and more compact SEI layer at 3 M compared to those formed at lower concentrations. Reproduced with permission [54]. Copyright 2018, American Chemical Society. (d) ^6^Li/^7^Li isotope‐exchange NMR intensity evolution over 75 h for (top) the ^7^Li metal signal and (bottom) the diamagnetic lithium signal (electrolyte + SEI) in LP30 (green) and LP30 + FEC (orange). The control experiment using natural‐abundance Li metal in LP30 + FEC is shown in purple. Reproduced with permission [98]. Copyright 2020, Royal Society of Chemistry.

LiFSI is often preferred over LiTFSI due to differences in the composition and quality of the SEI formed during reductive decomposition, as well as its greater compatibility with electrolyte design strategies. The reductive decomposition of the FSI^−^ anion initiates via S─F bond cleavage, which occurs spontaneously within 5.5 ps to generate LiF (Figure 6a) [1, 92]. The activation energy barrier for LiF formation via this pathway is relatively low (0.14 eV), resulting in a high LiF content within the SEI [54]. In contrast, LiTFSI preferentially undergoes C─S bond cleavage, which involves a higher energy barrier of 0.30 eV. Consequently, the –CF_3_ group in LiTFSI tends to form organofluorine‐rich phases within the SEI, which are detrimental to Coulombic efficiency [36]. XPS analyses reveal that SEI layers formed in both 1 M LiFSI/DME and 1 M LiTFSI/DME electrolytes consist of inorganic components, such as LiF and Li_3_N, as well as organic species, including ROCO_2_Li and ROLi. However, substantial differences are observed in their relative compositions (Figure 6b) [16]. In LiTFSI/DME, Li_3_N accounts for 50.33% of the inorganic lithiated species after 24 h of immersion, exceeding the LiF content. In contrast, Li_3_N is detected in LiFSI/DME only after 6 h of immersion.

Regulating the concentration of LiFSI represents a critical strategy for optimizing SEI physical properties. Variations in LiFSI concentration influence the lithium‐ion solvation structure, SEI formation behavior, and the resulting physical and chemical characteristics of the SEI. Wang, Muqin et al. systematically investigated the structural and compositional evolution of the SEI formed in a DME‐based LiFSI electrolyte by varying the salt concentration from 1 to 3 m, and demonstrated through SEM and XPS analyses that the SEI formed at 3 M LiFSI is thicker and more compact than those formed at lower concentrations (Figure 6c) [54]. In particular, the high‐concentration LiFSI electrolyte promotes preferential reductive decomposition of FSI^−^ anions, leading to a significantly increased LiF content and a higher fraction of inorganic components within the SEI, which collectively contribute to a denser interfacial structure. Increasing LiFSI concentration also promotes contact ion‐pair formation, in which Li^+^ ions interact more strongly with FSI^−^ anions than with solvent molecules, thereby lowering the LUMO energy of the anion. As a result, the anion becomes preferentially reduced at the Li metal surface, promoting anion‐derived decomposition pathways and facilitating the formation of an LiF‐rich solid electrolyte interphase [36].

Although direct modification of salt molecular structures, as exemplified by LiTFSI and LiFSI, can effectively enhance SEI stability, such approaches typically necessitate comprehensive redesign of the electrolyte system. While LiFSI improves SEI stability in ether‐based electrolytes, its combination with carbonate solvents—commonly employed in conventional LiPF_6_‐based electrolytes—may be unfavorable [57]. LiTFSI is generally paired with ether solvents (DOL, DME) rather than carbonate solvents (EC, DMC) owing to aluminum current collector corrosion concerns [23, 39, 45]. In contrast, additive‐based strategies, which enable precise interfacial chemistry control through the introduction of small quantities of functional molecules, offer a cost‐effective and readily applicable alternative.

#### 2.1.4 Interfacial Effects of FEC as a LiF‐Forming Additive

When a new lithium salt is introduced, the entire electrolyte system—including the solvent, salt concentration, and compatibility with the current collector—must typically be redesigned. Therefore, rather than replacing the entire electrolyte formulation, employing a small amount of functional additives to effectively regulate SEI formation represents a highly cost‐effective strategy that can be readily applied to existing systems, making it of considerable technical significance [45, 47]. Because additives are generally used at low volume fractions relative to the total electrolyte, electrochemical performance can be enhanced while preserving the primary composition of conventional LiPF_6_‐based carbonate electrolytes [28, 83].

Fluoroethylene carbonate (FEC) has been extensively investigated as one of the most representative and effective additives for generating LiF‐rich SEIs. The central role of FEC lies in its preferential reduction prior to the bulk solvent, which enables stable passivation of the lithium metal surface [69, 73, 100]. The reduction potential of Li^+^‐coordinated FEC lies in the range of 0.90–0.92 V, which is approximately 0.3 V higher than that of ethylene carbonate (EC), thereby favoring its preferential reduction [68, 98]. The reductive decomposition mechanism of FEC is thermodynamically highly favorable [69]. In the presence of Li^+^ ions, the reductive decomposition of FEC proceeds with a negligible activation barrier, rendering LiF formation nearly spontaneous [71].

The addition of FEC also significantly alters the local ionic environment within the electrolyte. Introducing 10 vol% FEC into a 1.0 M LiPF_6_/EC electrolyte increases the fraction of Li^+^–PF_6_^−^ contact ion pairs (CIPs) from 6% to 14% [69]. This increase in CIP population further promotes the reductive decomposition of PF_6_^−^ anions, thereby contributing to enhanced LiF formation.

Dynamic interfacial effects induced by FEC have also been quantitatively verified. According to ^6^Li/^7^Li isotope exchange nuclear magnetic resonance (NMR) measurements, the equilibrium lithium exchange flux between the lithium metal and the electrolyte approximately doubles upon FEC addition, increasing from 0.77 × 10^−^^6^ to 1.5 × 10^−^^6^ mol m^−^^2^ s^−^^1^ (Figure 6d) [98]. This enhancement indicates accelerated lithium‐ion transport across the FEC‐derived SEI, which may result in reduced overpotential and improved rate capability.

From a practical perspective, continuous recovery of the SEI following its initial formation is essential for ensuring long‐term cycling stability. Experimental studies suggest that a sufficiently high FEC concentration, typically ≥15 wt.%, is required to achieve effective lithium stabilization [101]. This observation implies that FEC is gradually consumed during electrochemical cycling. Although interface modification using small quantities of additives is an effective strategy, considerations regarding sustained SEI regeneration and overall electrolyte stability suggest that fluorination of the solvent itself—the primary component of the electrolyte—may provide a more fundamental solution.

However, although FEC is an effective additive for forming a LiF‐rich SEI, the gas evolution and parasitic reactions accompanying its reductive decomposition must also be carefully considered [91, 100]. When FEC undergoes reductive decomposition on the lithium metal surface, ring‐opening, defluorination, and decarboxylation reactions can proceed concurrently with LiF formation [102]. As a result, carbonate‐based gases such as CO_2_ and CO can be generated [71]. In addition, depending on the reaction conditions, side reactions accompanied by H_2_ evolution have also been reported [8, 26]. The ring‐opening and chain decomposition of FEC can be further accelerated at elevated temperatures or in the presence of Lewis acids, such as PF_5_ generated from LiPF_6_ decomposition [91]. Although such gas evolution may not be readily apparent at the coin‐cell level, it can cause increased internal pressure, cell swelling, deteriorated electrode–electrolyte contact, and interfacial delamination in large‐format pouch cells, thereby becoming a critical limitation for practical commercialization.

Therefore, in the design of FEC‐based electrolytes, FEC should not be used as a standalone additive without consideration of auxiliary additives and hybrid solvent compositions that can suppress gas evolution and parasitic reactions. For example, synergistic additives such as LiDFOB, LiBOB, LiFMDFB, and LiPO_2_F_2_ can complement the formation of the FEC‐derived LiF‐rich SEI and contribute to the development of a thinner, more uniform, and mechanically robust interphase [88, 103]. In addition, rather than excessively increasing the FEC content, an important strategy is to design hybrid electrolytes based on multiple fluorinated solvents, such as FEC/methyl (2,2,2‐trifluoroethyl) carbonate (FEMC)/hydrofluoroether (HFE) [9, 104]. In this configuration, FEC serves only the minimum role necessary for LiPF_6_ dissociation and initial LiF formation, while the remaining solvents are tailored to compensate for viscosity, oxidative stability, and suppression of gas evolution. Consequently, the effective use of FEC depends not simply on increasing its content, but on an optimized compositional design that simultaneously enables LiF‐rich SEI formation, suppresses gas evolution, mitigates electrolyte depletion, and controls interfacial resistance.

#### 2.1.5 Fluorinated Solvents for LiF‐Rich SEI Engineering

Fluorination of conventional solvent molecules represents a strategy that lowers the solvent LUMO energy to promote reductive decomposition and modulates the lithium‐ion solvation structure to induce anion decomposition, ultimately leading to the formation of a LiF‐rich SEI. The primary motivation for introducing fluorine atoms or fluorinated functional groups (e.g., –CF_3_) into solvent molecules is to tailor their electronic properties, rendering them more susceptible to reduction and facilitating LiF generation during decomposition [16, 98].

One representative approach involves substituting the terminal group of DME with a –CF_3_ group to form 1,1,1‐trifluoro‐2‐(2‐methoxyethoxy)ethane (TMEE) [57, 72]. Conventional ether solvents are relatively stable toward lithium metal but exhibit limited oxidative stability, which restricts their application in high‐voltage cathode systems. To address this limitation while promoting LiF‐rich SEI formation, terminal fluorination of ether solvents has been employed. The terminal –CF_3_ group exerts a strong electron‐withdrawing effect and displays lithiophobic behavior toward lithium ions, thereby reducing the binding energy between the solvent and Li^+^ (TMEE: −2.72 eV; DME: −2.84 eV). This weakened interaction promotes increased participation of anions in the Li^+^ solvation shell [57]. When combined with 2 M LiFSI salt, TMEE‐based electrolytes achieved Coulombic efficiencies exceeding 99.65%, attributable to the formation of a bilayer LiF‐rich SEI. An alternative approach involves substituting the terminal ethyl group of 1,2‐diethoxyethane(DEE) with –CF_3_ and –CF_2_ moieties. Yu, Zhiao, et al. reported that in electrolytes containing 1.2 M LiFSI dissolved in FnDEE solvents (*n* = 3, 4, 5, 6), LiF exhibited increased abundance and enhanced vertical uniformity in F4DEE and F5DEE systems incorporating terminal –CF_2_ groups [21]. Moreover, the –CF_2_ terminal group was found to possess a local dipole moment that interacts more strongly with lithium ions than the –CF_3_ group, as evidenced by shorter Li–F distances (–CHF_2_: 1.96 Å vs –CF_3_: 2.04 Å). In systems employing 1 M LiPF_6_ dissolved in solvents including FEC, 3,3,3‐fluoroethyl methyl carbonate(FEMC), and 1,1,2,2‐tetrafluoroethyl‐2′,2′,2′‐trifluoroethyl ether(HFE), LiF contents as high as ∼90% have been reported [9]. These electrolytes also exhibit excellent oxidative stability, with no detectable oxidative decomposition up to 6.5 V. In the case of 1 M LiFSI/2,2,2‐trifluoroethyl 3,3‐dimethylbutyrate(FDMB) electrolytes, a Coulombic efficiency of 99.4% was maintained after 300 cycles in Li/Cu cells [21, 45].

Overall, fluorinated solvents enhance the availability of fluorine species capable of forming LiF, thereby promoting the formation of LiF‐rich SEIs on lithium metal surfaces [86]. However, significant limitations accompany the design of fully fluorinated electrolyte systems. As solvent fluorination increases, viscosity generally rises while salt solvation capability decreases [21]. These changes can reduce ionic conductivity and adversely affect high‐rate charging performance [9, 105]. For instance, highly fluorinated ether solvents such as F6DEE exhibit poor ionic conductivity, resulting in degraded electrochemical performance. In contrast, partially fluorinated solvents incorporating –CHF_2_ groups at optimized levels may provide a more favorable balance between ionic conductivity and interfacial stability [21].

In addition, fluorinated solvents and lithium salts are considerably more expensive than conventional carbonate solvents and LiPF_6_ salts, potentially posing economic challenges for large‐scale manufacturing and commercialization [2, 45, 106]. Moreover, excessive LiF formation can be detrimental by thickening the SEI and reducing overall ionic conductivity [24, 53, 97, 107].

#### 2.1.6 Full‐Cell Interfacial Stabilization by Fluorinated Electrolytes

**FIGURE 7:**
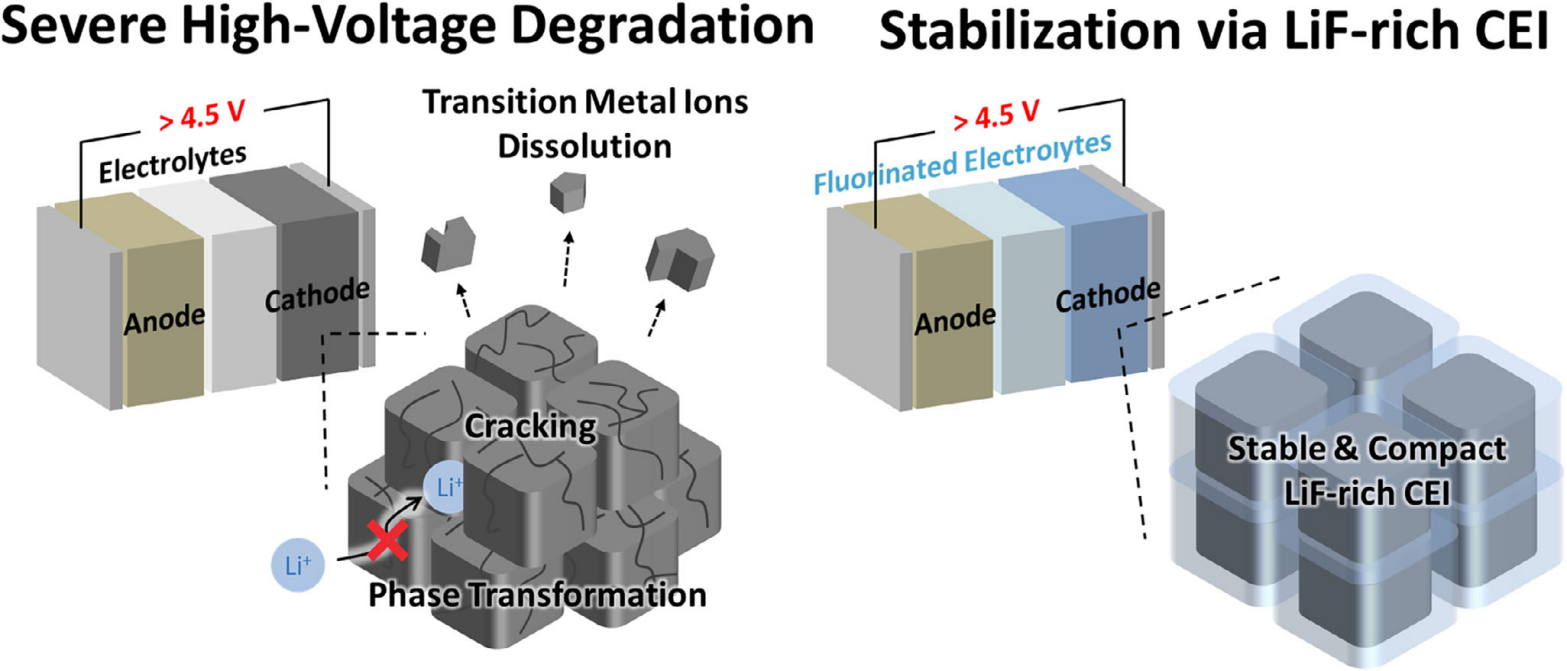
At high‐voltages above 4.5 V, the cathode surface undergoes severe degradation reactions, including the oxidative decomposition of the electrolyte, transition metal ion dissolution, phase transformation, and particle cracking. Fluorinated electrolytes enable the formation of a robust and dense LiF‐rich CEI, which effectively shields the cathode surface and mitigates structural collapse even under extreme high‐voltage conditions.

Existing studies on LiF‐rich SEIs have primarily focused on suppressing Li dendrite growth and stabilizing the SEI on the lithium metal anode. However, because practical LMBs operate as full‐cell systems in which the anode and cathode share the same electrolyte, the effects of fluorine‐based electrolytes are not limited to the anode interphase alone [45, 91]. In particular, high‐voltage cathodes such as Ni‐rich NCM and LiCoO_2_, which are widely employed in high‐energy LMBs, undergo complex degradation processes during charging, including oxidative electrolyte decomposition, lattice oxygen release, surface phase transformation, particle cracking, and transition metal dissolution (see Figure 7) [4, 88]. Therefore, the design of fluorine‐based electrolytes should be evaluated from a full‐cell perspective that simultaneously stabilizes both the SEI on the Li metal anode and the CEI on the cathode surface.

Under high‐voltage conditions, the cathode surface is exposed to intensive electrolyte oxidation [108]. In particular, at potentials above 4.5 V, lattice oxygen can be irreversibly released from the cathode surface, and the resulting oxygen‐deficient surface may undergo phase transformation from the original layered structure to spinel or rock‐salt phases with sluggish Li^+^ conductivity [109]. Such surface reconstruction increases the cathode/electrolyte interfacial resistance and obstructs Li^+^ transport pathways, thereby leading to capacity fading and power degradation [110]. In addition, anisotropic volume changes during repeated Li^+^ insertion and extraction induce intergranular cracking in polycrystalline cathode particles. The newly exposed surfaces generated by these cracks further react with the electrolyte, thereby accelerating interfacial degradation [88].

To mitigate such cathode degradation, fluorine‐based electrolytes can promote the formation of a LiF‐rich CEI on the cathode surface [99]. Under high‐voltage conditions, fluorine‐containing salts, solvents, and additives undergo oxidative decomposition to form a robust and dense CEI composed of organic and inorganic fluorinated species, including LiF, Li_x_PO_y_F_z_, Li_x_BO_y_F_z_, and CF_x_ [9, 91, 108]. This fluorine‐rich CEI blocks direct contact between the cathode surface and the electrolyte, suppresses continuous oxidative electrolyte decomposition, and mitigates cathode surface corrosion caused by acidic byproducts [89]. In particular, the LiF‐based inorganic CEI protects the cathode surface through its high chemical stability and extremely low solubility, suppresses the dissolution of transition metals such as Ni, Co, and Mn, and retards structural collapse into spinel or rock‐salt phases [39, 109].

In full‐cell configurations, CEI stabilization is closely coupled with the stability of the anode SEI. Transition metal ions dissolved from the high‐voltage cathode can migrate through the electrolyte and become deposited within the anode SEI, thereby compromising its electronically insulating nature and accelerating reductive electrolyte decomposition as well as active lithium depletion [23, 111, 112]. In other words, degradation initiated at the cathode can ultimately cause severe deterioration of the anode SEI [108, 113]. A stable CEI formed by fluorine‐based electrolytes suppresses cathode surface corrosion and transition metal dissolution, thereby indirectly but significantly improving the chemical stability of the anode SEI [88]. Accordingly, LiF‐rich CEI and LiF‐rich SEI should not be regarded as isolated protective layers, but rather as an interconnected interfacial stabilization network within the full‐cell system.

Moreover, fluorinated electrolytes influence not only the reductive and oxidative stability of the electrolyte, but also the Li^+^ solvation structure [21, 57]. Fluorine substituents generally modify the electronic structure of solvent molecules, tuning their highest occupied molecular orbital (HOMO)/LUMO energy levels and weakening the interaction strength between Li^+^ and the solvent [114, 115]. This weakened interaction enables anions to participate more actively in the Li^+^ solvation sheath, thereby promoting anion‐derived interfacial decomposition to form inorganic‐rich SEI and CEI [96]. These inorganic‐rich interphases effectively suppress electrolyte oxidation at the cathode while inducing uniform Li^+^ flux and stable lithium electrodeposition at the anode, thereby mitigating polarization growth and capacity fading of the full cell [45].

However, from the perspective of full‐cell operation and practical industrial application, fluorine‐based electrolytes still face several challenges. Excessive fluorination can lead to increased electrolyte viscosity, poor wettability, reduced salt solubility, and deteriorated ionic conductivity, all of which can cause severe performance degradation under fast‐charging and lean‐electrolyte conditions [21, 57]. In addition, high‐concentration electrolytes (HCEs) or highly fluorinated solvents may impose a substantial economic burden on commercialization because of their high cost [90, 109, 114]. Therefore, the design of fluorinated electrolytes should be based on a balanced approach that simultaneously considers interfacial stability, ionic conductivity, wettability, viscosity, and cost‐effectiveness, rather than simply maximizing the formation of a LiF‐rich SEI/CEI.

Particularly in large‐format cells, the spatial uniformity of in situ LiF‐rich SEI formation can become a critical processing limitation [116]. In high loading and thick electrodes, electrolyte penetration into the inner core region is restricted [90]. As a result, while a LiF‐rich SEI may form rapidly in the outer regions reached first by the electrolyte, LiF formation in the inner regions may be delayed or incomplete [117]. Such differences in interphase composition and thickness between the core and outer regions can induce localized resistance increases and concentrated Li^+^ flux, ultimately resulting in inhomogeneous lithium electrodeposition and premature dendrite growth [42, 103]. Therefore, to achieve a uniform LiF‐rich interphase in large‐format cells, parameters such as electrolyte viscosity and wettability, electrode pore structure, tortuosity, electrode‐tab design, and stack pressure must be optimized [7, 87].

A representative strategy for mitigating these issues is the design of LHCEs, which has recently attracted considerable attention [41]. LHCEs retain the ability of HCEs to form anion‐derived interphases while incorporating low‐viscosity diluents, such as 1,1,2,2‐tetrafluoroethyl‐2,2,3,3‐tetrafluoropropyl ether (TTE), to reduce the viscosity of the bulk electrolyte [118]. This approach preserves the contact ion pair (CIP) and aggregate (AGG) solvation structures characteristic of HCEs while simultaneously improving electrolyte penetration into the high‐tortuosity electrodes of large‐format cells [45]. Consequently, TTE‐based LHCEs can reduce the distribution gap of fluorinated components between the outer and core regions of the electrode, thereby enabling more spatially uniform formation of LiF‐rich SEI/CEI throughout the cell [1].

From the perspective of mass production, optimization of the formation protocol is also essential for stable interphase construction [26, 119]. The initial formation process is not merely an activation step, but can instead be regarded as an interphase templating process that determines the composition, thickness, and microstructure of the SEI/CEI maintained during subsequent long‐term cycling [17]. Appropriate medium to high current density conditions can enhance the ion exchange rate at the lithium metal–SEI interface and contribute to the formation of a denser SEI microstructure [46]. Because current and temperature distributions can vary spatially in large‐format cells, a formation protocol combining an initial wetting/rest step with stepwise current increments is required [8, 113]. Moreover, because temperature inhomogeneity within the cell during formation can cause spatial variation in interfacial reaction kinetics and SEI thickness, thermal management strategies are needed to minimize temperature differences across the entire cell [7, 23].

Ultimately, fluorine‐based electrolytes should be understood as a full‐cell‐level interphase control strategy that encompasses not only stabilization of the SEI on the lithium metal anode, but also CEI formation on high‐voltage cathodes, suppression of transition metal dissolution, and mitigation of cathode–anode cross‐talk [91, 108, 111]. This perspective extends LiF‐rich interphase research beyond anode‐centered protective‐layer design toward comprehensive electrolyte design that simultaneously stabilizes both the cathode and the anode.

### 2.2 Ex Situ Formation of LiF‐Rich SEI via Artificial Coating

The SEI, which is formed through spontaneous electrochemical reactions between lithium metal and conventional electrolytes with inherently dynamic and complex characteristics, generally exhibits a mosaic structure in which organic and inorganic components are heterogeneously mixed [20, 25, 30]. This heterogeneous architecture induces localized concentration of lithium‐ion flux, thereby promoting dendrite‐like, tree‐branch‐shaped lithium growth [35, 98]. Furthermore, lithium metal experiences nearly infinite volume changes during repeated charge–discharge cycles [30], and the mechanically fragile nature of the native SEI prevents it from accommodating such deformation, leading to frequent cracking or fracture [2, 35]. Once disrupted, the SEI exposes fresh lithium surfaces to the electrolyte, triggering continuous SEI reformation. This repetitive process results in irreversible consumption of both electrolyte and active lithium, ultimately constituting a primary cause of rapid battery performance degradation [16, 66].

As an alternative strategy to overcome the intrinsic dynamic instability of naturally formed SEI, a static artificial SEI approach—wherein a uniform and stable protective layer is intentionally constructed on the lithium metal surface—has attracted increasing attention. This strategy seeks to bypass complex interfacial chemical reactions and markedly enhance the stability of the lithium metal interface by forming a uniform, high‐purity LiF layer.

#### 2.2.1 Gas‐Phase Fluorination for Artificial LiF Interphases

**FIGURE 8:**
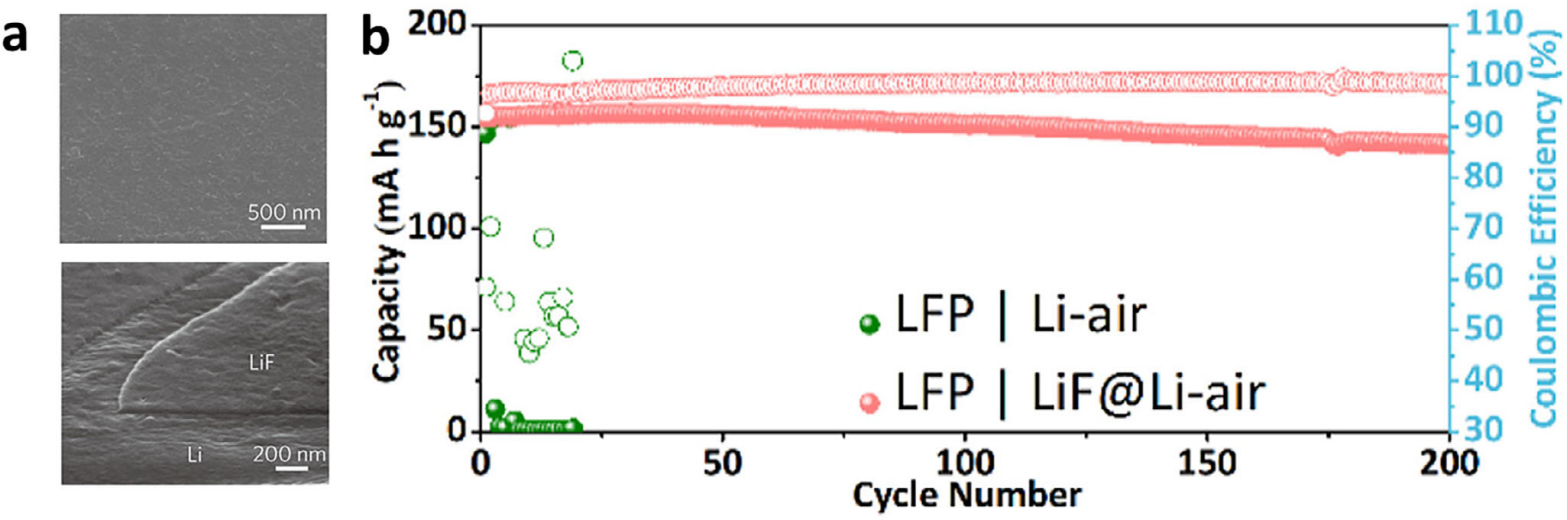
(a) Surface and cross‐sectional SEM images of LiF‐coated lithium foil prepared via gas‐phase fluorination, showing a smooth surface and a uniform LiF interphase formed on the Li metal surface. Reproduced with permission [55]. Copyright 2017, American Chemical Society. (b) Cycling performance and Coulombic efficiency of LiFePO_4_ full cells assembled with bare Li (LFP | Li‐air) and LiF‐coated Li (LFP | LiF@Li‐air) anodes. The LiF‐coated lithium anode exhibits significantly improved capacity retention and stable Coulombic efficiency during prolonged cycling. Reproduced with permission [120]. Copyright 2023, American Chemical Society.

Gas‐phase fluorination involves direct reaction between lithium metal and a fluorine‐containing gaseous medium to generate a protective LiF coating on the surface. This method enables the formation of a uniform coating through a solvent‐free, direct gas–solid reaction. In particular, LiF layers produced via the direct reaction between Freon R134a(1,1,1,2‐tetrafluoroethane) gas and lithium metal have been reported to significantly enhance interfacial stability [39, 55]. In one representative study, reaction at 150°C and 0.5 atm for 20 h resulted in the formation of an approximately 40 nm‐thick LiF layer (Figure 8a). This coating effectively suppressed initial SEI formation, exhibiting almost no impedance increase for 12 h following cell assembly. Moreover, the coated electrode demonstrated stable lithium deposition/stripping behavior for over 300 h at a current density of 1 mA cm^−^^2^ without a significant increase in overpotential. The lithium‐ion conductivity of the resulting LiF layer was estimated to be approximately 3 × 10^−^^9^ S cm^−^^1^. However, the air stability imparted by this coating was limited to only 900 s, highlighting challenges associated with practical process implementation.

An alternative gas‐based approach involves dissolving NF_3_ gas in an electrolyte and subsequently forming LiF through electrochemical reduction. In this method, a lithium–gas battery is assembled and discharged at a low current density of 1–10 µA cm^−^^2^, resulting in the deposition of LiF particles onto the lithium surface [24]. The net reaction produces only inert nitrogen gas (N_2_) as a gaseous byproduct, enabling ideal LiF formation without solid secondary products. X‐ray photoelectron spectroscopy (XPS) analysis confirmed a significant increase in fluorine content on the lithium surface after the reaction, while nitrogen content remained below 0.3%, verifying LiF as the nearly exclusive product [56]. Importantly, the total amount of deposited LiF and the particle size can be precisely controlled by adjusting the discharge capacity, providing a means to regulate particle size at the nanometer scale. Although gas‐based fluorination offers advantages in achieving uniform coatings, solvent‐based chemical approaches are also being actively explored due to the complexity associated with gas‐phase process control.

#### 2.2.2 Solvothermal Method

The solvothermal method involves heating lithium metal in fluorinated organic solvents at elevated temperatures within a sealed reactor to generate a LiF‐containing protective layer on the surface [120]. This approach features a relatively simple processing route while enabling the formation of a structurally complex SEI. The resulting SEI does not consist of a pure LiF layer but instead exhibits a composite structure comprising both inorganic (e.g., LiF, Li_2_CO_3_) and organic components. Detailed analyses reveal that inorganic species such as LiF are uniformly distributed throughout the entire SEI layer, whereas organic components form a heterogeneous outer layer concentrated within approximately 20 nm of the surface. Although this structure bears similarities to naturally formed SEI, its artificial origin may confer improved initial uniformity.

#### 2.2.3 Physical Vapor Deposition

Among physical vapor deposition (PVD) techniques, vacuum evaporation involves heating LiF under high‐vacuum conditions to generate a vapor phase, followed by its deposition onto a lithium metal surface to form a protective coating [39]. This deposition process avoids solution‐phase chemical reactions, enabling the formation of relatively high‐purity and homogeneous LiF layers with minimal impurity incorporation. It has been reported that a PVD‐derived LiF coating with a thickness of approximately 200 nm exhibits favorable electrochemical performance, maintaining stable lithium deposition at current densities up to 3 mA cm^−^^2^. In full cells employing a LiF@Li | LiFePO_4_ configuration, this artificial protective layer enabled a capacity retention of 85.31% after 600 cycles (Figure 8b). Additionally, stable lithium deposition and stripping were sustained for over 1200 h under lean electrolyte conditions (3 µL mg^−^^1^), highlighting the potential of PVD‐derived LiF coatings under relatively demanding operating conditions. Despite these advantages, such artificial coating strategies remain limited compared with the adaptive behavior of naturally formed SEI under dynamic battery operation.

#### 2.2.4 Fundamental Limitations of Artificial LiF Coatings

**FIGURE 9:**
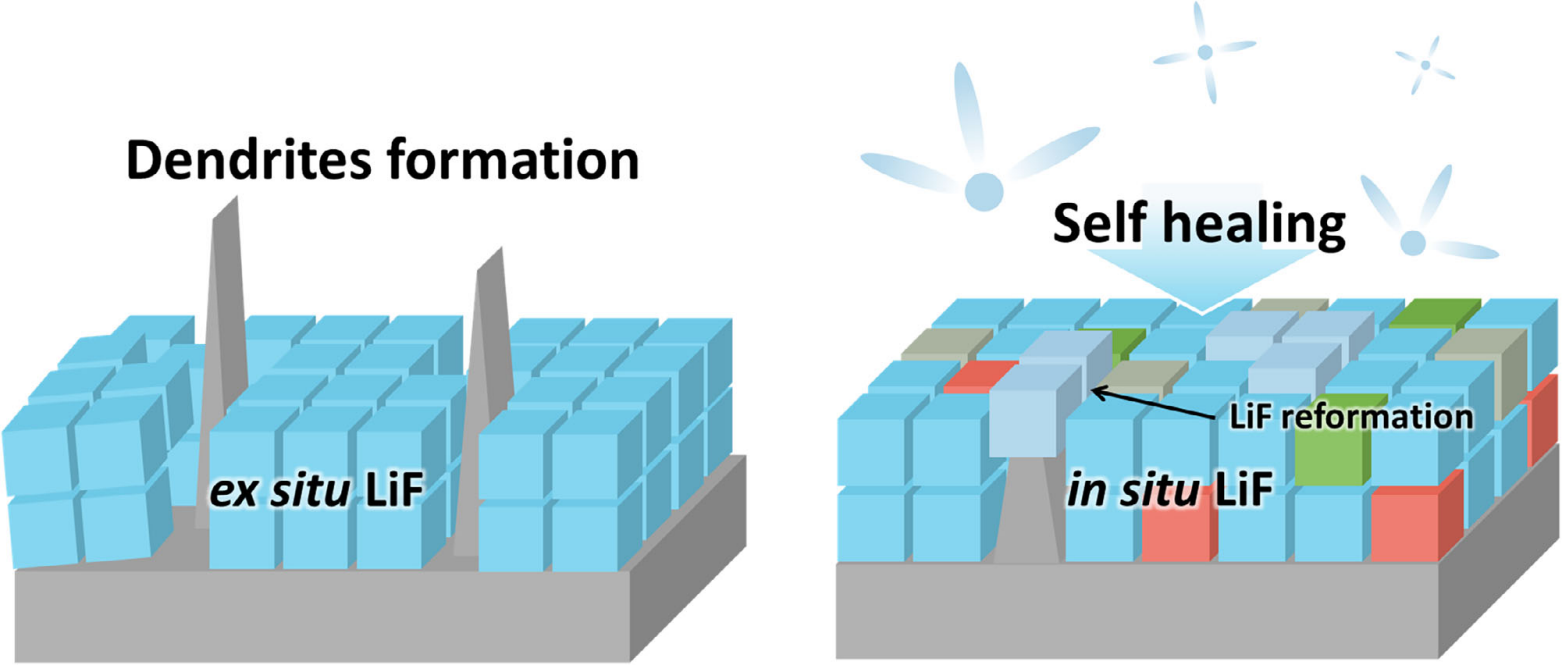
Schematic comparison of ex situ–formed LiF coatings and in situ–formed LiF‐rich SEI on lithium metal. Ex situ LiF layers lack regenerative capability once fractured, whereas in situ LiF‐rich SEI can be locally reformed through continued reduction of fluorine‐containing electrolyte species upon lithium exposure, enabling a self‐healing interphase.

Artificially fabricated SEIs offer a distinct advantage in that their thickness, purity, and uniformity can be precisely controlled during the design and fabrication stages. However, once formed, these coatings possess fixed physical properties, which limit their ability to adapt to the dynamically evolving lithium metal interface during repeated charge–discharge cycles. This fundamental constraint distinguishes artificial SEIs from naturally formed (in situ) SEIs, which continuously reorganize and partially repair themselves through ongoing electrolyte decomposition (see Figure 9).

The most critical limitation of artificial LiF coatings is their lack of self‐healing capability. Naturally formed SEIs exhibit dynamic behavior: when mechanical stress induces cracking during cycling, the surrounding electrolyte immediately decomposes to passivate and repair the damaged regions [111, 115]. In contrast, artificially deposited LiF layers lack this intrinsic self‐repair mechanism. Lithium metal undergoes theoretically infinite volume changes during cycling [30], and the substantial mechanical stresses generated readily induce cracking or catastrophic failure in the hard yet brittle artificial LiF layer. Once damaged, the coating cannot regenerate, leading to severe side reactions between the exposed lithium surface and the electrolyte and, consequently, rapid battery degradation.

Another fundamental limitation of artificial LiF coatings lies in their ionic transport properties. Most artificial coating strategies focus on forming single‐component LiF layers, whereas high‐performance in situ SEIs are intrinsically nanoscale composites composed not only of LiF but also of Li_2_O, organic species, and other components [20, 58]. Within these heterogeneous structures, interfaces between different phases—namely, heterogeneous grain boundaries—play a critical role in facilitating rapid lithium‐ion transport. At the LiF/Li_2_O interface, the binding energy of lithium ions is reduced, resulting in substantially higher ionic mobility compared to bulk phases [58, 121]. Moreover, the grain boundary energy of the LiF/Li_2_O interface (18.01 meV Å^−^^2^) is significantly lower than that of homogeneous LiF/LiF (23.80 meV Å^−^^2^) or Li_2_O/Li_2_O (34.08 meV Å^−^^2^) interfaces [6]. These quantitative results indicate that LiF/Li_2_O interfaces are thermodynamically more stable and provide energetically favorable pathways for ion migration. Consequently, artificial coatings composed solely of pure LiF monolayers inherently struggle to replicate the fast ion‐conduction pathways enabled by the complex nanostructure of naturally formed SEIs.

Although artificial LiF‐rich SEI coating strategies have demonstrated excellent interfacial stabilization at the laboratory scale, they face clear limitations in practical large‐scale manufacturing. In general, these coating approaches rely on F‐rich additives, highly concentrated fluorinated salts, fluorinated polymers or inorganic precursors such as AlF_3_ [37, 39]. Compared with conventional commercial electrolyte components or standard binders, these materials are associated with higher costs and more limited supply chains [90, 109, 114].

Furthermore, vapor‐deposition‐based processes such as atomic layer deposition (ALD) and PVD can produce uniform and defect‐free LiF thin films, but they require prohibitively expensive vacuum equipment and precursors [39, 115]. In addition, their inherently slow deposition rates make them unsuitable for the roll‐to‐roll continuous manufacturing needed for large‐area electrodes [46, 103]. Even in the case of solution‐casting or polymer‐based coating methods, multiple post‐treatment steps, including drying, thermal curing, and compression, are often required. The applicable temperature and pressure windows for these processes are also severely restricted by the intrinsically low melting point and high reactivity of lithium metal [35, 55].

Particularly in the fabrication of large‐format pouch cells, maintaining a uniform LiF‐rich layer with a thickness of only a few nanometers becomes extremely challenging, because spatial variations in coating thickness, applied pressure, and temperature become more pronounced as electrode area increases [7, 42, 115]. Therefore, for successful industrial implementation, process simplification strategies must be developed that simultaneously address material cost, process kinetics, equipment scalability, large‐area uniformity, and the thermal stability of lithium metal. Representative LiF‐rich SEI engineering strategies based on fluorinated electrolyte design and artificial interphase construction are compared in Table 1.

**TABLE 1:** Summary of representative LiF‐rich SEI engineering strategies and their electrochemical performance in lithium metal batteries.

Strategy / Method	Electrolyte Composition	Symmetric Cell Cycle Life	Capacity Retention	Coulombic Efficiency	SEI Components	Source
Fluorinated Solvent	1 M LiPF_6_ in FEC/FEMC/HFE (2:6:2)	500 cycles (approx. 2500 h)	90% after 450 cycles	99.20%	LiF, Li_2_O, PO_x_F_y_	[9]
Multisalt Electrolyte	0.225 M (LiFSI + LiPF_6_ + LiDFOB + LiTFSI) + 0.1 M LiNO_3_ in EC/DEC	—	95% after 200 cycles	99.10%	LiF, Li_2_O, Li_x_N	[121]
Countersolvent Electrolyte (TTE)	LiFSI in DMC:TTE (1:1)	1000 h at 1 mA cm^−2^	93.5% after 100 cycles at 0.5 C	98.6% average	LiF‐rich inorganic SEI	[106]
Fluorinated Additives	1 M LiPF_6_ in EC/EMC + 10 wt.% FEC	—	—	—	LiF, Li_2_CO_3_	[122]
Self‐Assembled Monolayers (SAMsC)	1.0 M LiTFSI + 0.2 M LiNO_3_ in DOL:DME (1:1) with Al_2_O_3_‐OOC(CH_2_)_2_COOH separator	2500 h	92.8% after 1000 cycles at 1 C	99.90%	LiF, Li_2_O, LiOH	[27]
Fluorinated Ether	1 m LiFSI in FDMB	—	—	—	LiF, Li_2_S, Li_2_O	[114]
Fluorinated Ether Solvent with FEC	1 M LiPF_6_ in FDMPr:FEC	—	80% after 520 cycles	99.85% average	LiF‐rich SEI	[10]
Trifluoromethylated DME Derivative (TMEE)	2 M LiFSI in TMEE	500 h	88% after 450 cycles	99.70%	Li_2_O	[57]
Fluorinated Cosolventization Diluent	LiFSI:DME:DFEB = 1:1.2:3 (molar ratio)	1000 h	85% after 650 cycles	99.90%	Li_2_O, LiF, LiOH, Li_2_CO_3_, Li_2_SO_4_	[41]
Fluorinated Solvent (FEC/FEMC)	1.2 M LiPF_6_ in FEC:FEMC (3:7 v/v)	—	73% after 400 cycles	99.9% average	LiF‐rich SEI	[105]
Surface Fluorination (Vacuum Evaporation)	1.0 mol L‐1 LiPF_6_ in EC/DEC (1:1 vol.)	800 h	85.31% after 600 cycles at 2 C	99.50%	LiF, Li_2_CO_3_, Li2O	[39]
High‐Concentration Full‐Fluoride Electrolyte	7 M LiFSI in FEC	300 h	78% after 130 cycles	99.64%	LiF, Li_2_CO_3_, Li_2_O	[11]
Catalytic Artificial SEI (Ni/C + PVDF)	1.0 M LiTFSI in DOL/DME (1:1) + 0.2 M LiNO_3_	1000 h at 10 mA cm^−2^	75.9% after 800 cycles	approx. 100%	LiF, Li_3_N, Li_2_CO_3_	[43]
Fluorinated Additive (EITC)	1.0 M LiPF_6_ in EC:DEC + 1 vol% EITC	600 h at 0.5 mA cm^−2^	81.4% after 1000 cycles at 10 C	99.1%	LiF, EITC polymer	[12]
Fluorinated Additives	1 M LiPF_6_ in EC/EMC + 10 wt.% FEC	—	—	—	LiF, Li_2_CO_3_	[122]
Artificial SEI (SnF_2_ Treatment)	1 M LiPF_6_ in EC/DEC (1:1) with SnF_2_	approx. 2325 h	80.01% after 150 cycles	approx. 99%	LiF, Sn, Li_5_Sn_2_	[31]
Highly Concentrated Carbonate	0.8 M LiFNFSI/FEBFP	>1500 h	500	99.3%	LiF‐rich SEI	[96]
Polar Polymer Coating (PAN‐b‐PSBMA)	1.0 M LiPF_6_ in FEC/DMC (1:4 v/v)	980 h at 0.5 mA cm^−2^	80% after 143 cycles	97.70%	LiF‐rich SEI	[49]
Fluorinated Carbon (CF_X_) Separator Coating	1 M LiPF_6_ in EC:DEC (3:7) + 10 wt.% FEC	290 h at 1 mA cm^−2^	86% after 150 cycles	—	LiF‐rich, Li_2_CO_3_	[123]
Solvothermal Treatment with FEC	1 M LiPF_6_ in EC/DMC (1:1)	500 h at 1 mA cm^−2^	90% after 200 cycles	98.60%	LiF, Li_2_CO_3_, ROCO_2_Li	[120]
Ionic Liquid Confined 3D MOF	PAN@ZIF/IL (EMIM‐TFSI + LiTFSI + LiNO_3_)	1000 h at 2 mA cm^−2^	95.3% after 500 cycles	approx. 99.9%	LiF, Li_3_N, Li_2_O	[124]

## 3 Dynamic Destruction and Reformation of the SEI

The SEI is often regarded as a static protective layer; however, in reality, it undergoes continuous destruction and reformation during repeated charge–discharge cycles [22, 65, 125]. This cyclic behavior indicates that the SEI is not merely a passive film, but rather a key component of a continuously evolving interfacial environment [126]. Accordingly, a detailed understanding of this dynamic behavior is essential for substantially improving battery lifetime and Coulombic efficiency (CE) [36, 115, 127].

The SEI formed on lithium metal anodes is subjected to extreme mechanical stress during electrochemical cycling [34, 52, 67]. This stress originates from the intrinsic volume changes associated with lithium metal plating and stripping, which can theoretically be regarded as infinite due to the continuous deposition of newly formed lithium [14, 23, 52, 67]. The freshly deposited lithium displaces or deforms the pre‐existing SEI layer, generating significant mechanical stress that ultimately results in SEI cracking or structural failure [22, 34, 111].

Hard inorganic SEI components, such as LiF, Li_2_O, and Li_2_CO_3_, are generally highly brittle [6, 30]. Although these inorganic phases exhibit high mechanical stiffness [30, 56], their low fracture toughness renders them vulnerable to the formation of microcracks under localized stress concentrations induced by volume fluctuations [6]. In particular, LiF‐rich SEIs can exhibit a high elastic modulus of up to 10.7 GPa, reflecting excellent mechanical rigidity [54]. In contrast, organic SEI components are mechanically soft and readily deform under applied stress [34]. Such deformability prevents effective stress redistribution and promotes the formation of localized “hot spots” where lithium‐ion flux becomes concentrated, thereby serving as preferential sites for dendritic lithium growth [128]. Consequently, the contrasting mechanical properties of inorganic and organic SEI constituents play a critical role in governing the overall mechanical response and failure behavior of the SEI.

When mechanical degradation leads to crack formation within the SEI, the highly reactive lithium metal surface is directly exposed to the electrolyte through these defects [30, 45, 127]. The exposed lithium surface immediately reacts with electrolyte components, initiating a re‐passivation process that either generates a new SEI layer or repairs the damaged region [56]. Although this process is essential for restoring interfacial stability, it is accompanied by the continuous consumption of active lithium and electrolyte species. Therefore, minimizing these parasitic losses requires that damaged regions be repaired as rapidly as possible, underscoring the importance of achieving a fast repassivation rate.

Electrolyte additives play a critical role in regulating this repassivation kinetics [47]. For instance, the addition of FEC to a conventional electrolyte composed of 1 M LiPF_6_ in EC/DMC increases the SEI formation rate by approximately fourfold [98]. Fluorinated electrolytes typically exhibit lower LUMO energies, enabling more rapid reactions with lithium metal to form thin and dense SEI layers. This characteristic facilitates rapid SEI repair when the pre‐existing SEI is disrupted.

## 4 Characteristics and Control Strategies of LiF in the SEI

The SEI faces a fundamental dilemma in that it must simultaneously and effectively fulfill two inherently contradictory roles [18, 64, 129]. First, the SEI must function as an electronic insulator that completely blocks electron migration to the lithium metal surface [22, 36], thereby suppressing continuous electrolyte decomposition and ensuring long‐term battery stability and lifetime. Second, the SEI must provide highly efficient transport pathways for lithium ions (Li^+^), effectively acting as a low‐resistance highway that enables smooth and rapid charge–discharge processes [20, 52]. In other words, the SEI must exhibit high ionic conductivity to support fast electrochemical kinetics [28, 129].

Within these competing requirements, LiF—one of the key inorganic constituents of the SEI—occupies a unique position, as it represents both the core challenge and a potential solution [20, 24, 36]. LiF exhibits the most outstanding electronic insulating properties among SEI components [106]; however, it intrinsically suffers from extremely low ionic conductivity [97, 130]. This duality clearly indicates that the performance of LiF‐rich SEIs is not determined solely by the amount of LiF present, but is instead governed by the nanoscale structure of the SEI and its interactions with coexisting components [6, 20, 30, 56, 58]. Accordingly, this review aims to elucidate the intrinsic limitations of bulk LiF and to propose design principles for optimized SEIs through precise structural and compositional control.

### 4.1 Electronic Insulation of LiF

#### 4.1.1 Wide Bandgap and Intrinsic Electronic Insulation

The most prominent electronic characteristic of LiF is that it possesses the widest bandgap among known SEI components [131]. The bandgap is defined as the minimum energy required for an electron to transition from the valence band to the conduction band; a larger bandgap therefore corresponds to more effective electronic insulation. Although reported bandgap values for LiF vary depending on experimental and computational methodologies, they consistently indicate exceptionally large values, including 8.9 [45, 131]. and 13.6 eV [11, 24].

These values are substantially higher than those of other major inorganic SEI components, such as Li_2_CO_3_ (≈4.75 eV) and Li_2_O (≈4.7 eV), demonstrating that LiF is, in principle, the most effective electronic insulator among SEI constituents [45, 131, 132]. This wide bandgap is directly correlated with the extremely low electronic conductivity of LiF, which is on the order of 10^−^^1^ ^3^–10^−^^14^ S cm^−^^1^, effectively suppressing electron transport through the SEI layer [28, 72]. In addition, LiF exhibits a broad electrochemical stability window ranging from 0 V to 6.4 V versus Li/Li^+^, enabling it to maintain chemical stability even under high‐voltage operating conditions [24].

#### 4.1.2 Critical Thickness for Suppressing Electron Tunneling

In nanometer‐thick SEI layers, the dominant mechanism responsible for electron leakage is the quantum mechanical phenomenon of electron tunneling [23, 34, 65]. Electron tunneling refers to the ability of electrons to penetrate energy barriers that are classically forbidden, owing to the probabilistic nature of their wave functions [18]. Within the SEI, electrons are unable to classically overcome the energy barrier between the Fermi level of lithium metal and the conduction band minimum of SEI components; nevertheless, a finite tunneling probability allows a weak electronic current to pass through sufficiently thin SEI layers.

$$T=\exp\left(-2\frac{\sqrt{2m\Delta E}}{h}d\right)$$

When the SEI thickness d is small, the tunneling probability does not decay sufficiently, allowing electrons to penetrate the SEI layer. As the SEI grows and reaches a critical thickness, the tunneling probability decreases exponentially, effectively suppressing further electrolyte reduction reactions [5, 65]. As a result, SEI growth becomes self‐limiting [65, 133]. In this expression, T denotes the tunneling probability, m is the electron mass, ΔE represents the barrier height determined by the energy gap and Fermi level offset, d is the SEI thickness, and h is Planck's constant.

The electronic insulating capability of an SEI is therefore dictated by its ability to suppress electron tunneling [65]. Because the electronic structures of SEI constituents—particularly their bandgaps and tunneling barriers (ΔE)—vary significantly, the minimum thickness required to effectively block tunneling depends strongly on the SEI composition. Among major inorganic SEI components, LiF requires the smallest critical thickness to suppress electron tunneling [34]. Specifically, the minimum thicknesses required to block tunneling are reported as follows: LiF, 1.652 nm; Li_3_PO_4_, 1.75 nm; and Li_2_CO_3_, 2.13 nm. This comparison indicates that LiF can achieve effective electronic insulation at substantially thinner thicknesses. Such a thin critical thickness is advantageous for minimizing lithium consumption during SEI formation, thereby reducing the initial irreversible capacity loss [34, 65].

#### 4.1.3 Influence of LiF Microstructure on Electronic Insulation

While single‐crystalline LiF represents the ideal electronic insulator in theory, SEIs formed in practical battery systems are inherently polycrystalline aggregates [24, 64]. The key distinction arises from the presence of grain boundaries, which are nanoscale interfaces between crystalline domains and serve as structural and electronic discontinuities that fundamentally compromise the insulating integrity of the SEI layer [64, 134].

Grain boundaries in polycrystalline LiF exhibit significantly higher electronic conductivity than bulk single‐crystalline LiF. Density functional theory (DFT) calculations reveal that the electrical conductance of LiF containing grain boundaries is more than an order of magnitude higher than that of defect‐free LiF [134]:

Conductivity of pure LiF: 4.35 × 10^−^^5^ G_0_

Conductivity of LiF with grain boundaries: 8.28 × 10^−^^4^ G_0_

This enhanced conductivity originates from a localized reduction in the bandgap at grain boundary regions [64]. Grain boundaries with narrowed bandgaps can therefore serve as preferential pathways for electron transport, effectively acting as electron leakage channels [34]. These localized conduction paths can, in turn, promote lithium dendrite growth, posing a serious threat to interfacial stability [23, 111]. Importantly, this phenomenon is not unique to LiF; defects and grain boundaries in solid electrolytes represent a general issue that can facilitate unintended electronic conduction [6, 34].

In addition to grain boundaries, atomic‐scale defects and chemical or structural inhomogeneities within the SEI can also contribute to electron leakage [20, 111]. Although an ideal SEI is expected to exhibit self‐limiting growth and stabilize at a thickness of only a few nanometers, naturally formed SEIs are often observed to reach thicknesses of several tens of nanometers [17, 54]. This discrepancy cannot be explained by electron tunneling alone and suggests that persistent electron leakage through grain boundaries and defects overrides the self‐limiting behavior of the ideal SEI [133]. Such defect‐mediated electron transport drives continuous electrolyte reduction, leading to excessive SEI thickening and ultimately resulting in irreversible capacity loss [31, 34].

### 4.2 Ionic Conductivity

A careful examination of the physical properties of LiF reveals a fundamental limitation: LiF alone cannot constitute an ideal SEI [36, 56]. This limitation originates from its extreme electrochemical characteristics—namely, its outstanding electronic insulating capability combined with intrinsically poor ionic conductivity [131, 135].

The diffusion energy barrier for Li^+^ migration within the LiF crystal lattice is reported to be as high as 0.729 eV, which is significantly larger than that of other major inorganic SEI components, such as Li_2_O (0.152 eV) and Li_2_CO_3_ (0.227–0.491 eV) [131]. As a direct consequence of this high migration barrier, the ionic conductivity of LiF is extremely low, on the order of ∼3 × 10^−^^9^ S cm^−^^1^ [55]. This indicates that bulk LiF crystals behave as near‐perfect insulators not only for electrons but also for lithium ions, thereby introducing a severe bottleneck for Li^+^ transport and potentially limiting rate capability and fast‐charging performance in lithium metal batteries [29, 131].

Based on these considerations, it becomes evident that an SEI composed of a thick, uniform, single‐component LiF layer could effectively suppress electron leakage but would simultaneously impede Li^+^ transport, ultimately resulting in degraded electrochemical performance [37, 52]. Therefore, the design strategy for SEIs in high‐performance lithium metal batteries should move beyond simply increasing the LiF content and instead focus on precisely controlling the structure and spatial distribution of LiF to secure efficient ion‐conduction pathways [36, 53, 57].

#### 4.2.1 Interface Engineering Strategies for Enhancing Ionic Conductivity

**FIGURE 10:**
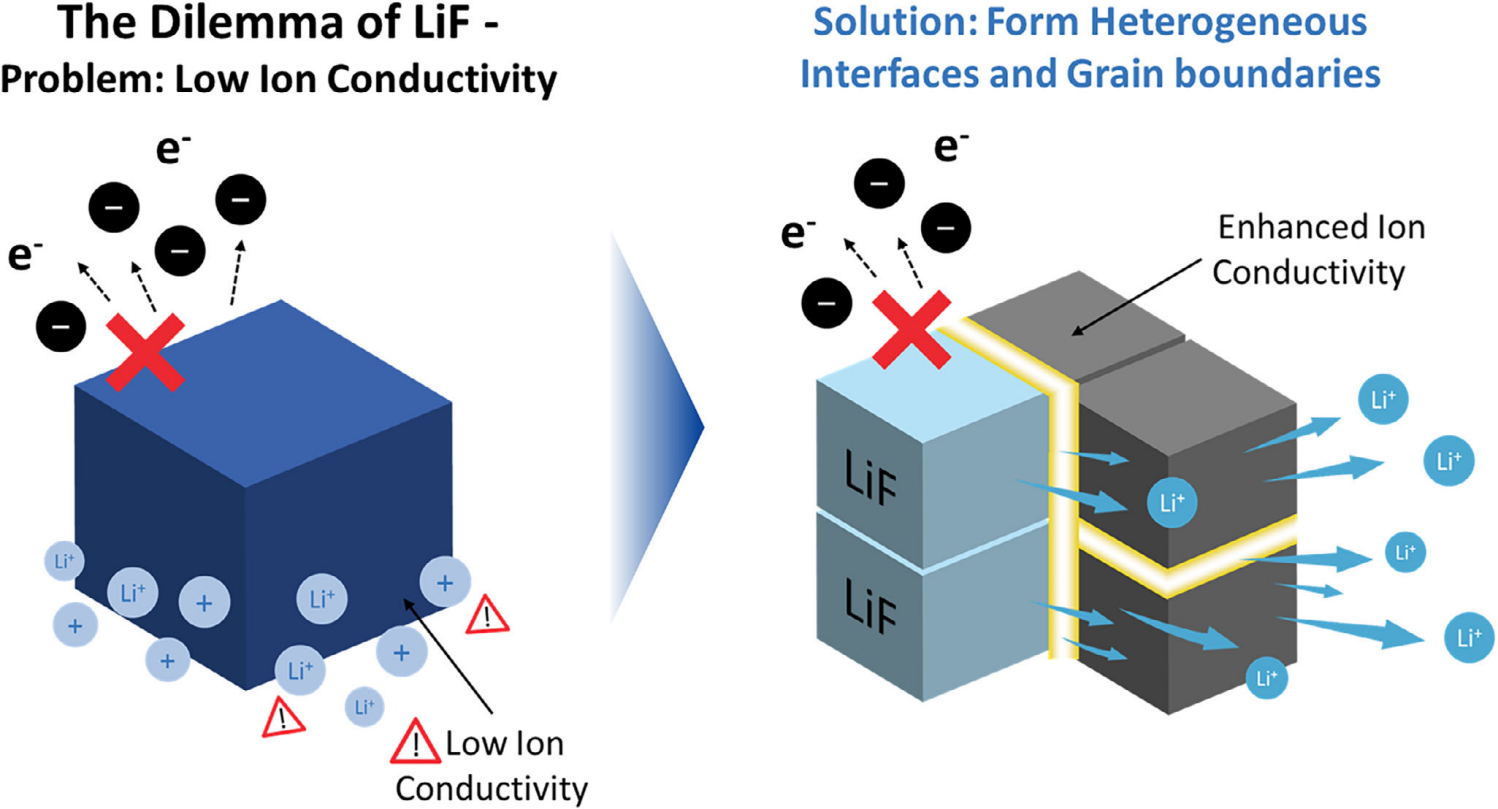
Schematic illustration of interface engineering to overcome the intrinsically low ionic conductivity of bulk LiF. A homogeneous bulk LiF phase severely restricts Li^+^ transport due to slow lattice diffusion, whereas the introduction of heterogeneous interfaces and grain boundaries within a LiF‐based SEI creates energetically favorable fast pathways for Li^+^ migration while preserving strong electronic insulation.

A key strategy for overcoming the inherently low ionic conductivity of LiF discussed above is interface engineering (see Figure 10) [36]. This approach is based on creating alternative Li^+^ transport pathways that bypass the slow diffusion processes within the bulk LiF crystal lattice [52, 58]. By intentionally enriching the SEI with heterogeneous interfaces and grain boundaries, so‐called “fast pathways” can be introduced, along which Li^+^ ions can migrate with significantly reduced energy barriers. Such nanoscale structural control represents one of the most promising strategies for substantially enhancing the overall ionic conductivity of the SEI while preserving the excellent electronic insulating properties of LiF [37].

Ionic conductivity is markedly enhanced at heterogeneous interface regions where distinct inorganic phases meet [111, 126, 128]. In particular, the LiF/Li_2_O heterointerface exhibits higher Li^+^ mobility than either bulk LiF or bulk Li_2_O alone [58]. This behavior can be attributed to changes in the local coordination environment of Li^+^ ions at the interface. Whereas Li^+^ ions are fully and stably coordinated within an ideal bulk crystal, the incomplete coordination environment at the interface renders the ions structurally and energetically unstable. This coordination frustration reduces the binding energy of Li^+^, thereby lowering the activation barrier for site‐to‐site hopping and resulting in enhanced ionic mobilit. In other words, the interfacial region constitutes an energetically more accessible landscape for Li^+^ transport. Given that Li^+^ binding interactions in bulk inorganic crystals are extremely strong—approximately seven times stronger than in lithium metal itself—such interfacial “shortcuts” become particularly critical for enabling efficient ion transport [20, 131].

The formation of heterogeneous interfaces such as LiF/Li_2_O is also thermodynamically favored. The grain‐boundary energy of the LiF/Li_2_O heterogeneous interface is reported to be 18.01 meV Å^−^^2^, which is substantially lower than that of homogeneous LiF/LiF (23.08 meV Å^−^^2^) or Li_2_O/Li_2_O (34.08 meV Å^−^^2^) interfaces [6]. This energetic preference indicates that heterogeneous interfaces are more stable than homogeneous ones, providing a thermodynamic driving force for the spontaneous formation of heterogeneous nanocrystalline structures during SEI formation [58, 64].

Multi‐salt electrolyte systems represent a practical example of how interfacial engineering can be effectively implemented [45]. Unlike single‐salt electrolytes, which tend to produce relatively uniform SEIs, multi‐salt electrolytes contain multiple anions (e.g., FSI^−^, TFSI^−^, PF_6_^−^). Upon electrolyte decomposition, these systems simultaneously generate a variety of inorganic SEI components, including LiF, Li_2_O, Li_3_N, and Li_2_S [23, 111, 121]. The coexistence of these phases leads to the formation of nanoscale grains with abundant grain boundaries and heterogeneous interfaces within the SEI [132]. These interfaces collectively form a percolated network of high‐speed Li^+^ transport pathways, significantly enhancing the overall ionic conductivity of the SEI [121]. This concept of improving ionic conductivity through deliberate interface control provides a critical framework for defining the optimal structural and compositional characteristics of LiF‐rich SEI [36, 53].

#### 4.2.2 From Bulk Barrier to Interfacial Li^+^ Transport Network

Interface engineering strategies become even more critical under practical cell operating conditions, particularly under fast‐charging or extreme‐temperature environments [53, 136]. Although bulk LiF provides excellent electronic insulation and chemical stability, its intrinsically low Li^+^ conductivity can become a severe kinetic bottleneck under high‐current‐density conditions [44]. During fast charging, a large amount of Li^+^ must pass through the SEI and reach the lithium metal surface within a short period [46, 52]. If Li^+^ transport within the SEI is not sufficiently rapid, local ion concentration gradients and interfacial polarization develop, leading to concentrated Li^+^ flux at specific regions and thereby promoting non‐uniform lithium nucleation and dendrite growth [72, 98]. Therefore, in a high‐performance LiF‐rich SEI, LiF should not simply exist as a bulk protective phase, but should be precisely organized at the nanoscale to provide low‐resistance Li^+^ transport pathways.

A key advantage of nanoscale LiF lies in its ability to reduce the effective migration distance across low‐ionic‐conductivity bulk LiF regions [24, 72]. When LiF exists as a thick and continuous layer, Li^+^ must diffuse directly through the LiF lattice and thus experiences substantial diffusion resistance [41, 44]. In contrast, when LiF is uniformly dispersed as fine nanocrystals or nanoparticles, Li^+^ can preferentially migrate along particle surfaces, grain boundaries, and surrounding heterointerfaces [132]. In such an architecture, LiF retains its electron‐blocking capability, while the surrounding interfacial network serves as a fast pathway for Li^+^ transport [37]. Especially under fast‐charging conditions, these continuous and uniform interfacial transport pathways alleviate local ion depletion and current concentration, thereby suppressing localized vertical dendritic growth [96, 137].

Recent studies have shown that combining LiF with an inorganic phase possessing high Li^+^ conductivity is effective for improving high‐rate performance [124]. In particular, LiF/Li_3_N‐based interphases have attracted considerable attention because they can simultaneously exploit the electronic insulation and chemical stability of LiF, together with the high Li^+^ conductivity and lithiophilic nature of Li_3_N [40, 42]. In such structures, LiF acts as a protective phase that suppresses electron leakage and continuous reductive decomposition of the electrolyte, whereas the Li_3_N‐rich regions or LiF/Li_3_N heterointerfaces function as conductive interlayers that facilitate rapid Li^+^ transport [44, 137]. This indicates that, unlike a single‐component LiF layer, which increases interfacial resistance under high‐current conditions, a LiF‐based interphase can support fast Li^+^ transport through synergistic coupling with surrounding phases [36, 70].

The spatial control of nanoscale LiF is also a critical factor governing fast‐charging performance [53]. Confining LiF nanoparticles within an aligned polymer matrix, porous structure, or laminate‐type artificial interphase can suppress their random agglomeration and establish uniform Li^+^ transport pathways [138]. In such cases, Li^+^ migrates not by directly penetrating the highly resistive bulk LiF regions, but rather along nanoparticle surfaces or polymer/inorganic interfaces [44, 132]. This spatially confined architecture uniformly distributes Li^+^ flux throughout the SEI and mitigates localized Li over‐deposition, which is prone to occur during fast charging [63, 137]. In other words, the nano structuring of LiF should be understood not merely as a change in interphase composition, but as a strategy for redesigning the Li^+^ transport pathways themselves.

Interphase‐driven ion transport is also essential from the perspective of dendrite suppression. At high current densities, Li^+^ near the electrode surface is rapidly consumed, and if ion supply through the SEI cannot keep pace, local concentration polarization develops [2]. In this case, an SEI composed of nanoscale LiF connected by heterointerfaces enhances the effective migration rate of Li^+^, thereby mitigating interfacial concentration gradients and preventing current concentration at specific sites [24]. As a result, lithium electrodeposition is more likely to proceed uniformly along the surface as planar growth rather than vertical dendritic growth [9]. Thus, nanoscale LiF should be regarded not merely as a passive protective byproduct, but as an active structural element that regulates the spatial distribution of Li^+^ flux during high‐rate operation.

Nanoscale LiF and heterointerface‐driven transport are also important across a wide temperature range. At low temperatures, increased electrolyte viscosity, higher Li^+^ desolvation barriers, and sluggish diffusion within the SEI occur simultaneously, resulting in a substantial increase in interfacial resistance [91]. Under such conditions, a bulk‐LiF‐dominated SEI can further deteriorate low‐temperature performance because of its poor ionic conductivity [53]. In contrast, in a LiF‐rich SEI in which grain boundaries and heterointerfaces are interconnected, Li^+^ can migrate along interfacial pathways with relatively lower energy barriers rather than directly penetrating the LiF lattice, thereby alleviating low‐temperature polarization [44, 58]. Under high‐temperature conditions, although electrolyte decomposition and SEI reconstruction are accelerated, the high chemical stability and low solubility of LiF are advantageous for maintaining a protective interphase [33, 139]. Therefore, a LiF‐rich SEI that operates stably over a wide temperature range must simultaneously incorporate thermally stable LiF domains and continuous fast Li^+^ interfacial transport pathways.

The low ionic conductivity of bulk LiF is a kinetic limitation that must be addressed in the design of LiF‐rich SEIs [41]. However, rather than using LiF as a thick single‐phase protective layer, this limitation can be substantially alleviated by engineering LiF into nanoparticles, nanocrystals, heterointerfaces, polymer/inorganic hybrid pathways, and interconnected grain‐boundary networks. Particularly under fast‐charging and extreme‐temperature conditions, structural designs that enable Li^+^ to migrate rapidly along interfaces rather than through bulk LiF are essential [53]. Accordingly, future research on LiF‐rich SEIs should move beyond simply increasing LiF content and instead focus on the precise control of LiF domain size and spatial distribution, interfacial connectivity with surrounding phases, and the continuity of Li^+^ transport pathways.

### 4.3 Controversial Roles and Control Strategies of LiF

Recent advances in advanced analytical techniques have introduced new perspectives that challenge the traditional understanding of the role of LiF in the SEI. These perspectives can be broadly categorized into two opposing viewpoints. The conventional view considers LiF to function as a key protective component within the SEI owing to its intrinsically favorable physicochemical properties, including a wide electrochemical stability window (0–6.4 V vs Li/Li^+^), a large bandgap, and high surface energy [11, 20, 24]. From this perspective, increasing the LiF content in the SEI is regarded as directly contributing to enhanced battery performance [140].

In contrast, an emerging viewpoint suggests that LiF exists in the actual SEI as discontinuous nanoparticles and is structurally unstable, thereby limiting its ability to function as an intrinsic protective layer [24, 51]. According to this perspective, the performance improvements observed in LiF‐rich SEIs arise not from the inherent properties of LiF itself, but rather from synergistic interactions with other SEI components or from additional, less apparent mechanisms [31, 36, 58].

**FIGURE 11:**
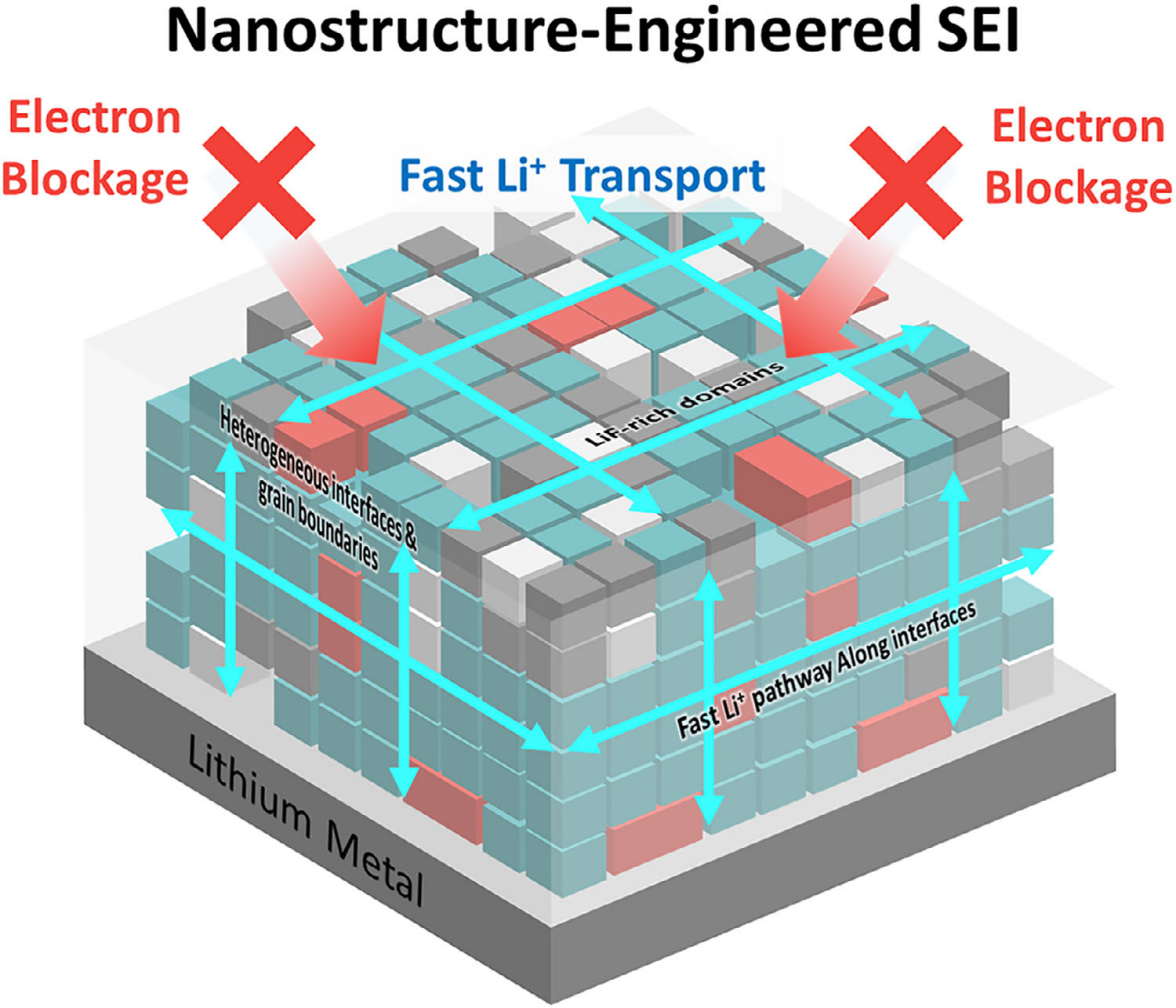
Schematic illustration of a nanostructure‐engineered SEI design concept that simultaneously satisfies electronic insulation and fast Li^+^ transport. Electronically insulating LiF blocks suppress electron leakage, while a network of heterogeneous interfaces and grain boundaries provides continuous fast ion‐conduction pathways, enabling LiF to function as a controllable building block rather than a homogeneous protective layer within the SEI.

This evolving understanding underscores the fact that the SEI is not a single, homogeneous phase, but instead a complex and dynamic composite system [17, 31, 56, 132]. Resolving this controversy does not require choosing one viewpoint over the other; rather, it necessitates a multidimensional perspective that regards LiF not as an isolated protective layer, but as a controllable building block that participates in constructing the SEI nanostructure (see Figure 11) [20, 53].

#### 4.3.1 Intrinsic Limitations of LiF‐Based Protection

Advanced analytical techniques, particularly cryogenic transmission electron microscopy (cryo‐TEM), have enabled direct visualization of nanometer‐scale SEI structures at near‐atomic resolution, which was not possible using conventional characterization methods [3, 23, 111]. Traditionally, LiF was assumed to form a dense, continuous film that uniformly covers the lithium metal surface [51]. However, numerous cryo‐TEM studies have revealed that LiF does not form a uniform layer, but instead exists as nanoparticles that are discontinuously dispersed within the SEI matrix [27, 51]. Such a discontinuous nanoparticle morphology fundamentally compromises one of the core functions of the SEI—namely, electronic insulation [56]. The gaps between LiF nanoparticles provide pathways through which electrons can continuously leak to the lithium metal surface, sustaining electrolyte reduction reactions, destabilizing the SEI, and accelerating electrolyte consumption [34]. In addition, this discontinuous structure is mechanically weaker than an idealized continuous film, making it more vulnerable to disruption by the substantial volume changes that occur during lithium plating and stripping. As a result, fresh lithium surfaces are repeatedly exposed to the electrolyte, further exacerbating interfacial instability [45]. Thus, regardless of LiF's excellent intrinsic insulating properties, its effectiveness is inherently limited if it cannot form a structurally continuous membrane [36, 51, 71].

The intrinsic electrical properties of LiF also introduce fundamental challenges in SEI engineering. When the LiF layer thickness exceeds approximately 2 nm in an effort to suppress electron leakage, the resulting high ionic resistance inevitably leads to severe overpotential (polarization) during lithium plating and stripping, thereby degrading battery efficiency and cycle life [34]. Conversely, reducing the LiF layer thickness to lower ionic resistance fails to sufficiently suppress electron tunneling, allowing electrolyte decomposition to persist. This issue is further amplified by the discontinuous nanoparticle morphology discussed above. Even when individual LiF particles exceed the critical thickness of ∼2 nm, the gaps between particles effectively reduce the local thickness to zero, creating direct pathways for electron leakage [51, 134].

Taken together, the conflict between structural discontinuity and electrochemical requirements indicates that LiF alone cannot serve as an ideal protective interphase. However, this discontinuous nanoparticle morphology should not be regarded simply as an unavoidable intrinsic limitation of LiF itself [51]. In practical SEIs, LiF is formed under highly non‐equilibrium electrochemical conditions, where rapid electrolyte reduction, local supersaturation of LiF‐forming species, heterogeneous nucleation, particle growth, and incomplete coalescence collectively determine the final morphology [141]. Therefore, the discontinuous distribution of LiF should be understood as a kinetic outcome of SEI formation rather than merely as a material‐intrinsic defect [142]. From this perspective, the key challenge in LiF‐rich SEI design is not only to compensate for the limitations of LiF, but also to actively regulate its nucleation and growth dynamics [24]. These observations indicate that the SEI design approach must shift away from simply increasing the amount of LiF and toward precise control over the formation pathway and nanostructure of the entire SEI, including LiF and its interfaces‐particularly grain boundaries and heterogeneous interfaces‐with other inorganic components [26, 64].

#### 4.3.2 Nanostructural Control of LiF‐Rich SEI

Despite the limitations of LiF discussed above, LiF‐rich SEIs remain among the most promising strategies for achieving high‐performance lithium metal batteries. Importantly, this promise does not arise from the properties of LiF as an isolated material, but rather from its ability to form favorable nanostructures through synergistic interactions with other SEI components [30, 37, 53, 121, 135]. Accordingly, the key to successful SEI design lies not in simply increasing the LiF content, but in optimizing its morphology, spatial distribution, and interfacial relationships with surrounding components [20, 57].

An effective SEI must simultaneously satisfy two inherently conflicting requirements: rapid lithium‐ion transport and efficient suppression of electron transfer [34]. The most effective structural solution to this dilemma is one in which LiF exists not as a thick, heterogeneous layer, but as a thin, dense, and uniformly dispersed nanocrystalline phase [24, 36]. Such a configuration minimizes the thickness required to suppress electron tunneling while also reducing the overall diffusion distance for Li^+^ ions, thereby mitigating increases in ionic resistance [11, 17]. The SEI itself is a heterogeneous composite with a thickness of only several nanometers, formed through a series of extremely rapid and complex interfacial reactions [114]. As a result, purely experimental approaches face inherent limitations in fully resolving the dynamic formation mechanisms and atomic‐scale structure of the SEI [111]. In this context, molecular dynamics (MD) and DFT‐based simulations provide indispensable tools for overcoming these limitations, offering critical atomic‐level insights into LiF formation processes and enabling rational strategies for controlling SEI nanostructure [1, 3, 38, 71].

#### 4.3.3 Atomistic and Computational Strategies for LiF‐Rich SEI Engineering

The SEI formed at the lithium metal electrode–electrolyte interface is an extremely thin layer, typically only a few nanometers thick, and it develops over an exceptionally short timescale ranging from picoseconds to nanoseconds [24, 133, 143]. Owing to these characteristics, fundamental limitations exist in experimentally tracking the initial nucleation stage of the SEI and the associated atomic‐level chemical reactions in a continuous manner. Despite the advent of advanced analytical techniques such as X‐ray absorption spectroscopy, in situ transmission electron microscopy (TEM), and atomic force microscopy (AFM), most experimental approaches provide only temporally segmented, static information in the form of snapshots. As a result, directly elucidating the continuous reaction pathways at the interface and the real‐time behavior of transient intermediates remains highly challenging [16, 142].

**FIGURE 12:**
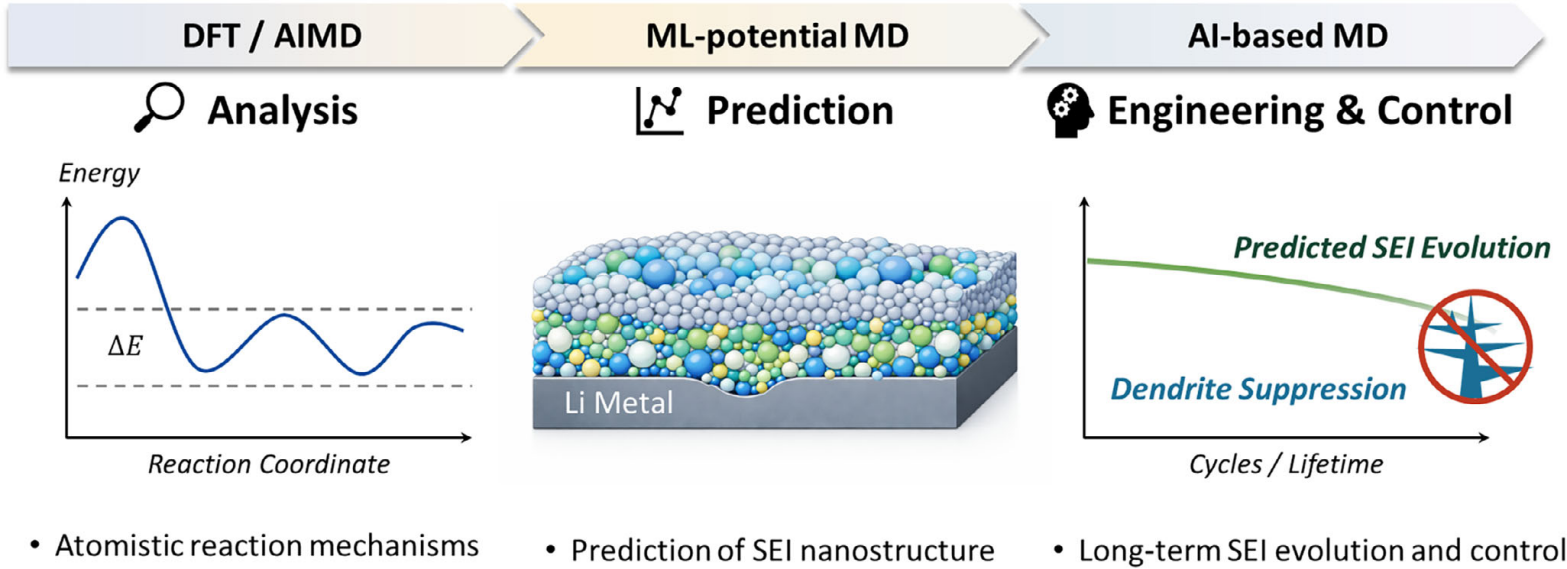
Conceptual illustration of molecular dynamics–based computational strategies for SEI design and prediction. DFT and AIMD elucidate elementary interfacial reactions and LiF formation mechanisms, while machine‐learning potential and AI‐based molecular dynamics extend simulations to larger systems and longer timescales, enabling prediction of SEI evolution and lithium dendrite suppression.

To overcome these experimental limitations, computational science approaches based on MD and DFT have emerged as essential tools for elucidating SEI formation mechanisms occurring in the nanometer–picosecond regime, which is largely inaccessible by experiment alone (see Figure 12) [58, 144]. Computational simulations offer a unique capability to directly trace, at the atomic level, how electrolyte molecules are reduced and decomposed on the lithium metal surface and through which reaction intermediates they are subsequently transformed into SEI components, including LiF [28, 145, 146].

DFT plays a central role in revealing the fundamental principles governing SEI formation by quantitatively predicting the energetics of specific chemical reactions and the electronic structure of materials based on quantum mechanical theory [58, 142, 144]. In particular, DFT enables rigorous evaluation of the thermodynamic feasibility of SEI component formation by calculating free‐energy profiles and activation energy barriers along entire reaction pathways [71, 133, 141]. For example, in analyses of the one‐electron reduction decomposition of the electrolyte additive FEC, DFT calculations have demonstrated that cleavage of the C─O bond, rather than simple C─F bond scission, is the energetically most favorable pathway for LiF formation when transition‐state energetics are explicitly considered [71]. Such results provide strong mechanistic evidence explaining why specific additives preferentially form LiF‐rich SEIs through well‐defined atomic‐scale reaction pathways, rather than through empirical correlations alone.

Beyond additive decomposition, DFT is also widely employed to compare and assess competing reaction pathways through which lithium salts, such as LiFSI, decompose to yield LiF[54], as well as to accurately predict the reduction potentials of solvent, salt, and additive molecules constituting the electrolyte [68, 114]. For instance, DFT calculations have revealed that FEC exhibits a reduction potential approximately 0.3 V higher than that of the primary solvent, ethylene carbonate (EC) [71]. This theoretical prediction directly supports experimental observations showing that FEC is preferentially reduced prior to EC, leading to the formation of an initial LiF‐rich SEI layer that enhances interfacial stability [69]. In addition, DFT can be used to evaluate the mechanical stability and adhesion of the SEI by calculating the binding energy between SEI components and the lithium metal surface [6].

Molecular dynamics simulations complement DFT by elucidating how these interfacial reactions evolve along the real time axis [3, 45, 73]. In particular, AIMD explicitly propagates atomic motion according to quantum mechanical forces, enabling direct observation of chemical reactions as they occur over time at the atomic scale [1, 57]. For example, during the reduction of fluorine‐containing additives such as FEC on lithium metal surfaces, AIMD simulations can capture the entire reaction cascade—including bond cleavage and recombination events—occurring within extremely short timescales of several hundred femtoseconds (10^−^^1^ ^5^ s), ultimately leading to the nucleation and growth of LiF nanocrystals [28, 71, 73].

Computational simulations also provide critical insights for interpreting complex experimental data. In the case of intricate nuclear magnetic resonance (NMR) spectra obtained from reduction products of ^1^ ^3^C‐labeled FEC additives, simulations can be used to calculate theoretical spectra for plausible intermediate and product species, such as vinoxyl radicals. Direct comparison between simulated and experimental spectra enables reliable assignment of subtle spectral features and unambiguous identification of previously unresolved chemical species [32]. This highlights that simulations serve not merely as auxiliary tools for validating experimental observations, but as indispensable approaches for resolving analytical challenges that are difficult to address experimentally.

Despite its high accuracy, traditional AIMD is inherently limited by its substantial computational cost, restricting simulations to systems containing at most several thousand atoms and timescales on the order of hundreds of picoseconds [144, 145]. To address these limitations, machine‐learning potentials (MLPs) have recently gained significant attention. By training on DFT‐generated datasets, MLPs can retain near‐DFT accuracy while enabling simulations of much larger systems—comprising tens of thousands to hundreds of thousands of atoms—over extended timescales exceeding hundreds of picoseconds [147]. Using such approaches, SEI formation at the lithium metal/β‐Li_3_PS_4_ solid‐state electrolyte interface has been visualized at atomic resolution. Simulations involving approximately 12,000 atoms successfully elucidated the mechanisms of SEI growth and stabilization, with the predicted final SEI thickness (≈12 nm) quantitatively matching high‐resolution TEM observations. These results confirmed a dual‐layer structure consisting of crystalline Li_2_S regions adjacent to the lithium metal and amorphous regions toward the electrolyte side [145].

Nevertheless, even MLP‐based molecular dynamics simulations remain computationally demanding for directly simulating the tens to hundreds of charge–discharge cycles corresponding to practical battery lifetimes. To bridge this remaining timescale gap, AI‐based atomistic forecasting approaches have recently been proposed. These methods leverage existing MD datasets to predict long‐term atomic‐scale evolution beyond the reach of conventional simulations. By simultaneously forecasting structural evolution and electrochemical state variables, such models achieve computational accelerations of tens of times or more—reducing calculations that previously required tens of hours to only tens of minutes—while maintaining quantitative agreement with experimentally observed structural descriptors and morphological trends. This capability suggests that AI‐based prediction represents a promising computational strategy for efficiently capturing long‐term SEI evolution and dendrite growth behavior [143].

Although these simulations offer the advantage of providing atomic‐level reaction mechanisms, it is difficult for them to fully capture the complexity, interfacial heterogeneity, and spatiotemporal scale limitations of practical battery environments [144]. On the other hand, while experimental analysis can directly verify the composition, morphology, and spatial distribution of the SEI formed after practical battery operation, it faces limitations in continuously elucidating the initial reaction pathways that lead to electrolyte decomposition and inorganic phase formation [145]. Therefore, recent studies have actively adopted a complementary approach that combines computation and experiment to overcome the limitations of each method and cross‐validate the SEI formation mechanisms [38]. Cheng et al. analyzed the onset potential and thickness variations of the SEI by tracking the depth profile of interfacial ion signals using in situ liquid secondary ion mass spectrometry (SIMS), directly observed the actual thickness and morphology of the formed SEI using cryo‐TEM, and compared the reduction stability of the solvation complexes via DFT calculations [62]. As a result, they revealed that differences in the Li^+^ solvation structure of the electrolyte govern not only the onset potential and final thickness of the SEI but also the dynamic evolution of the inner and outer SEI. Zhang, Xingxing, et al. proposed through DFT and AIMD/MD simulations that TFSI^−^ and NO_3_^−^ coordinate more strongly with Li^+^ than the solvents do, and are preferentially reduced and decomposed to form LiF and Li_3_N [124]. Based on this, they performed SEM, cryo‐TEM, electron energy loss spectroscopy (EELS)/ Energy Dispersive X‐ray Spectroscopy (EDS) mapping, XPS, and TOF‐SIMS analyses, confirming that a LiF/Li_3_N‐rich Janus interphase was formed on the nanofiber surface under the PAN@ZIF/IL condition, effectively suppressing dendrite growth. These results demonstrate that the anion‐derived decomposition tendency is linked to the actual interphase formation, while simultaneously suggesting that its formation site and structure are highly influenced by the ZIF pore confinement and Lewis acid sites. These cases demonstrate that the integration of computation and experiment goes beyond a simple comparison of results, acting as a robust verification framework that links the causes and consequences of SEI formation. DFT and MD explain processes that are difficult to directly observe experimentally‐such as electrolyte decomposition pathways, electron transfer, ion coordination structures, and inorganic phase nucleation—at the atomic level, whereas experimental analyses like cryo‐TEM, SEM, and XPS structurally and chemically verify whether these predictions are realized in practical battery environments [23]. In the study of LiF‐rich SEIs, this computation–experiment cross‐validation strategy extends beyond merely identifying SEI compositions; it serves as a core methodology to elucidate why a specific electrolyte design forms a stable interphase and, ultimately, to predictively design SEIs with desired compositions and nanostructures [34, 133].

Taken together, this computational framework—spanning DFT, MD, machine‐learning potentials, and AI‐based predictive models—functions as a powerful design platform that extends beyond retrospective interpretation of experimental results. Instead, it enables the proactive design and optimization of LiF‐rich SEIs with targeted properties. In particular, molecular dynamics‐based simulations facilitate the a priori prediction of structural and property changes in the SEI induced by variations in electrolyte composition, thereby allowing efficient exploration of novel electrolyte systems capable of delivering optimized electrochemical performance.

## 5 Conclusion

Lithium metal batteries have attracted significant attention as next‐generation high‐energy storage systems owing to their high theoretical capacity and low reduction potential. However, their commercialization remains fundamentally constrained by the instability of the SEI formed at the lithium metal–electrolyte interface [7]. In particular, the heterogeneous nature of the SEI promotes lithium dendrite growth, continuous electrolyte depletion, and low Coulombic efficiency. To address these challenges, the formation of a LiF‐rich SEI has been proposed as a key strategy [15, 26, 35, 39]. In this review, LiF‐rich SEI is not regarded merely as a passive protective layer, but rather as a dynamic interfacial complex whose performance is governed by its nanostructure and interfacial characteristics. Accordingly, the principles underlying its formation and control have been systematically examined.

LiF‐rich SEIs can be realized either through in situ formation using fluorinated salts, additives, and solvents in the electrolyte, or through ex situ approaches based on artificial surface coatings [98]. Electrolyte‐based strategies naturally generate multiple inorganic components, including LiF, leading to the spontaneous formation of nanoscale crystallites and heterogeneous interfaces within the SEI. In contrast, artificial coating strategies provide high initial uniformity and compositional purity but suffer from limited adaptability to dynamic electrochemical environments and a lack of intrinsic self‐healing capability [16]. This comparison highlights that SEI performance is dictated not simply by the presence of LiF, but by how LiF is incorporated into the SEI nanostructure and by the nature of its interfaces with other inorganic and organic components.

The SEI is inherently a dynamic interfacial structure that undergoes repeated destruction and reformation during repeated lithium plating/stripping cycles. Extreme volume changes associated with lithium metal plating and stripping impose substantial mechanical stresses on the SEI [47, 125, 130]. Inorganic components such as LiF exhibit high stiffness but are intrinsically brittle, rendering them susceptible to cracking, particularly along grain boundaries and heterogeneous interfaces [5, 6, 11, 14]. Once damage occurs, electrolyte decomposition resumes locally and new SEI layers form. In this context, grain boundaries and heterogeneous interfaces function not merely as structural imperfections, but as critical elements that govern both SEI reformation dynamics and lithium‐ion transport behavior [45, 58, 121]. Consequently, SEI stability is determined not by the mechanical properties of any single component, but by the collective response of the entire nanostructure to coupled mechanical and electrochemical stresses [57].

Among SEI components, LiF provides the most effective electronic insulation; however, in its bulk form it suffers from a fundamental drawback—extremely low ionic conductivity [11, 56, 131]. Thick LiF layers severely impede Li^+^ transport, whereas excessively thin layers fail to fully suppress electron tunneling. In practical SEIs, LiF does not exist as a continuous film but rather as discontinuous nanocrystalline domains [134, 135]. The grain boundaries and heterogeneous interfaces associated with this morphology can act as electron leakage pathways, while under appropriate conditions also serving as fast Li^+^ transport channels [58, 121, 134]. As a result, recent SEI control strategies have shifted away from approaches that rely solely on LiF as a protective layer and toward the deliberate design of nanocomposite SEIs in which LiF coexists with other inorganic phases such as Li_2_O and Li_3_N, enabling controlled utilization of grain boundaries and heterointerfaces.

In summary, achieving stable lithium metal batteries requires moving beyond simply maximizing the formation of a LiF‐rich SEI and advancing toward nanostructure‐control strategies that enable efficient Li^+^ transport while preserving the electron‐blocking capability of LiF. In practical SEIs, LiF does not form a continuous protective film, but is instead distributed as nanocrystals. The grain boundaries and heterointerfaces formed in this configuration serve as key structural elements that determine the balance between electron‐blocking capability and Li^+^ transport. Therefore, future design of LiF‐rich SEIs must move beyond merely increasing LiF content and instead focus on precisely tailoring the size and spatial distribution of LiF domains, grain‐boundary density, heterointerface characteristics, and continuous Li^+^ transport pathways.

To achieve this goal, a prediction‐driven research strategy based on simulation‐guided interphase design is essential. LiF‐rich SEIs are formed within a short timescale (ps–ns) in an interphase layer only a few nanometers thick, representing a highly complex system in which electrolyte decomposition, LiF nucleation, electron transfer, Li^+^ diffusion, and grain‐boundary formation occur simultaneously. These initial reactions and nanostructure evolution processes are extremely difficult to track directly by experiment. Therefore, DFT, AIMD, and MLP‐based molecular dynamics should not be used merely as auxiliary tools for interpreting experimental results, but rather as core methodologies for prospectively elucidating SEI formation mechanisms and proposing interphase design strategies.

In particular, simulations can predict which electrolyte–interphase combinations are capable of simultaneously satisfying electronic insulation and facile ionic transport by precomputing and comparing electrolyte compositions, fluorinated solvent structures, lithium salt decomposition pathways, and Li^+^ migration barriers at heterointerfaces. Furthermore, MLPs and AI‐based molecular dynamics can extend the spatiotemporal reach of conventional DFT/AIMD, enabling the evaluation of LiF spatial distribution, grain‐boundary connectivity, SEI growth behavior, and dendrite nucleation propensity in much larger interfacial models. Accordingly, future computational studies should evolve beyond descriptive analyses of pre‐formed SEIs into predictive platforms capable of prospectively designing LiF‐rich SEIs with targeted compositions and nanostructures, while also screening promising electrolyte candidates.

Ultimately, research on LiF‐rich SEIs should be expanded into a robust framework in which simulations first predict the electrolyte–interphase structure, experimental analyses validate these predictions, and the resulting insights are fed back into the computational models. Such an approach will minimize trial‐and‐error electrolyte development and enable the precise design not only of LiF content, but also of its distribution, grain boundaries, heterointerfaces, and Li^+^ transport pathways. Consequently, predictive interphase engineering grounded in computation–experiment integration will serve as a key strategy for accelerating the design of LiF‐rich SEIs for next‐generation high‐energy, high‐safety lithium metal batteries.

## Author Contributions

**Gwangsik Kim**: investigation, conceptualization, data curation, formal analysis, validation, visualization, resources, writing – original draft, writing – review and editing. **JinHyeok Cha**: conceptualization, data curation, investigation, validation, supervision, funding acquisition, project administration, resources, writing – review and editing.

## Conflicts of Interest

The authors declare no conflicts of interest.

## Data Availability

The data that support the findings of this study are available from the corresponding author upon reasonable request.
